# Supplementary material for: Effects of turmeric (Curcuma longa) supplementation on glucose metabolism in diabetes mellitus and metabolic syndrome: An umbrella review and updated meta-analysis
Source: PLoS One. 2023 Jul 20;18(7):e0288997. doi: 10.1371/journal.pone.0288997 (PMC10359013; doi:10.1371/journal.pone.0288997)
Supplement: S1 File — (ZIP) [file pone.0288997.s002.zip › Fig S17.pdf]

**Fig S17. Graphical Representation of Overlap for OVErviews (GROOVE) (14 meta-analysis included studies)**

## Overall results

| | | |
|---|---|---|
| Number of columns (number of reviews) | c | 14 |
| Number of rows (number of index publications) | r | 62 |
| Number of included primary studies (including double counting) | N | 155 |
| Covered area | N/(rc) | 17.86% |
| Corrected covered area | (N-r)/(rc-r) | 11.54% |
| Interpretation of overlap | High overlap | |
| Structural Zeros | X | 0 |
| Corrected covered area (adjusting by structural zeros) | (N-r)/(rc-r-X) | 11.54% |
| N° of non-overlapped primary studies | In 1 SR | 35 |
| Number of overlapped primary studies | In 2 SRs | 5 |
| | In 3 SRs | 5 |
| | In 4 SRs | 6 |
| | In 5 SRs | 3 |
| | In 6 SRs | 3 |
| | In 7 SRs | 2 |
| | In 8 SRs | 2 |
| | In 9 SRs | 0 |
| | In 10 SRs | 0 |
| | In 11 SRs | 0 |
| | In 12 SRs | 0 |
| | In 13 SRs | 0 |
| | In 14 SRs | 0 |
| | In 15 or more SRs | 0 |

## Graphical Representation of Overlap for OVErviews (GROOVE)

| | Zheng ZH, 2021 | Panahi Y, 2015 | Altobelli E, 2021 | de Melo, ISV, 2018 | Huang J, 2019* (only MetS, T2DI | Tabrizi R, 2018 | Ashtary-Larky D, 2021 | Azhdari, M.,2019 | Yuan, F., 2019 | Yuan, F., 2022 | Zhang, T, 2021 | Gorabi AM, 2022 | Macena ML, 2022 |
|---|---|---|---|---|---|---|---|---|---|---|---|---|---|
| Panahi Y, 2015 | 0.0% | | | | | | | | | | | | |
| Altobelli E, 2021 | 25.0% | 0.0% | | | | | | | | | | | |
| de Melo, ISV, 2018 | 5.6% | 0.0% | 20.0% | | | | | | | | | | |
| Huang J, 2019* (only MetS, T2DM) | 10.0% | 0.0% | 23.5% | 56.3% | | | | | | | | | |
| Tabrizi R, 2018 | 13.3% | 2.9% | 13.8% | 37.0% | 48.1% | | | | | | | | |
| Ashtary-Larky D, 2021 | 6.3% | 0.0% | 6.7% | 5.3% | 4.5% | 2.9% | | | | | | | |
| Azhdari, M.,2019 | 0.0% | 6.7% | 0.0% | 20.0% | 10.5% | 10.0% | 0.0% | | | | | | |
| Yuan, F., 2019 | 22.2% | 4.5% | 23.5% | 38.9% | 47.4% | 53.8% | 4.5% | 16.7% | | | | | |
| Yuan, F., 2022 | 19.0% | 4.0% | 20.0% | 33.3% | 55.0% | 43.3% | 4.0% | 14.3% | 55.0% | | | | |
| Zhang, T, 2021 | 42.9% | 0.0% | 18.8% | 4.5% | 8.3% | 11.8% | 5.0% | 0.0% | 18.2% | 16.0% | | | |
| Gorabi AM, 2022 | 15.4% | 14.3% | 16.7% | 0.0% | 5.0% | 6.5% | 0.0% | 16.7% | 10.5% | 14.3% | 11.8% | | |
| Macena ML, 2022 | 8.3% | 0.0% | 0.0% | 0.0% | 0.0% | 3.3% | 7.7% | 0.0% | 5.6% | 4.8% | 6.3% | 0.0% | |
| Tian J, 2022 | 21.4% | 0.0% | 14.3% | 0.0% | 9.5% | 9.4% | 0.0% | 0.0% | 15.0% | 18.2% | 23.5% | 33.3% | 7.7% |

| 91 | = | Total nodes (pairs of reviews) |
|---|---|---|
| 32 | = | Slight overlap (<5%) |
| 16 | = | Moderate overlap (5% to <10%) |
| 12 | = | High overlap (10% to <15%) |
| 31 | = | Very High overlap ($\geq$15%) |
