# Supplementary material for: Effects of turmeric (Curcuma longa) supplementation on glucose metabolism in diabetes mellitus and metabolic syndrome: An umbrella review and updated meta-analysis
Source: PLoS One. 2023 Jul 20;18(7):e0288997. doi: 10.1371/journal.pone.0288997 (PMC10359013; doi:10.1371/journal.pone.0288997)

Fig S18. Forest plot of difference in changes of post-intervention value and change from baseline for fasting blood glucose (FBG).

Study	Patients	Trial	Model	Mean difference (95% CI)	I^2
**FBG (mean difference, SMD)**					
Huang, J, 2019	T2DM	5	random-effect	-0.68 (-1.07, -0.29)	74.7
Tabrizi R, 2018	T2DM	7	random-effect	-1.09 (-1.91, -0.27)	94.8
Zhang, T, 2021	T2DM	5	random-effect	-0.28 (-0.62, 0.06)	72
Huang, J, 2019	NAFLD/MetS	8	random-effect	-0.15 (-0.35, 0.05)	78

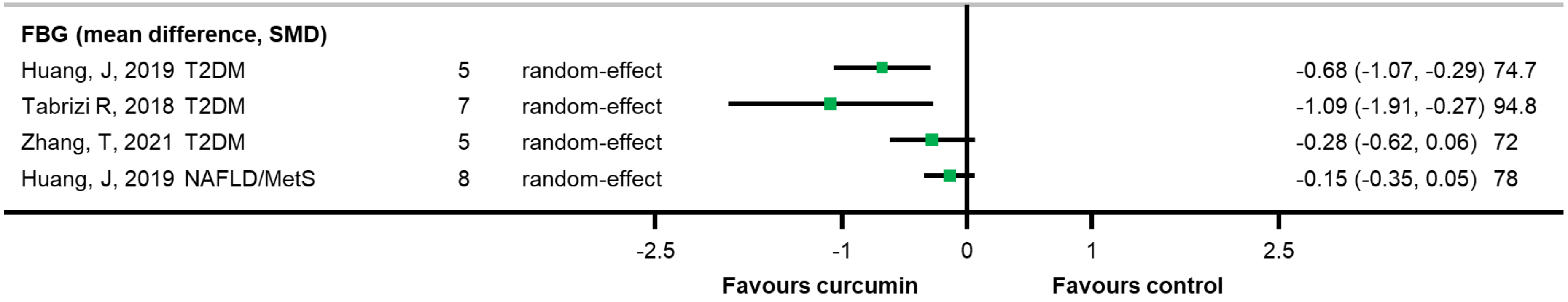

Study	Patients	Trial	Model	Mean difference (95% CI)	I^2
**FBG (mean difference, UMD)**					
Ashtary-Larky, D, 2021	MetS	3	random-effect	-28.29 (-63.34, 6.76)	68.7
Azhdari, M., 2019	MetS	5	random-effect	-9.18 (-16.70, -1.66)	90.1
Ashtary-Larky, D, 2021	T2DM	3	random-effect	-27.07 (-39.61, -14.52)	54.4
Tian J, 2022	T2DM	9	random-effect	-8.85 (-14.40, -3.29)	41.2
Yuan, F., 2022	T2DM	11	random-effect	-12.88 (-17.49, -8.28)	80
**FBG (post value, UMD)**					
de Melo, 2018	pre-DM, T2DM, MetS	8	random-effect	-13.86 (-21.17, -6.56)	74
Zheng ZH, 2021	T2DM	4	fixed-effect	-14.49 (-21.20, -7.79)	34

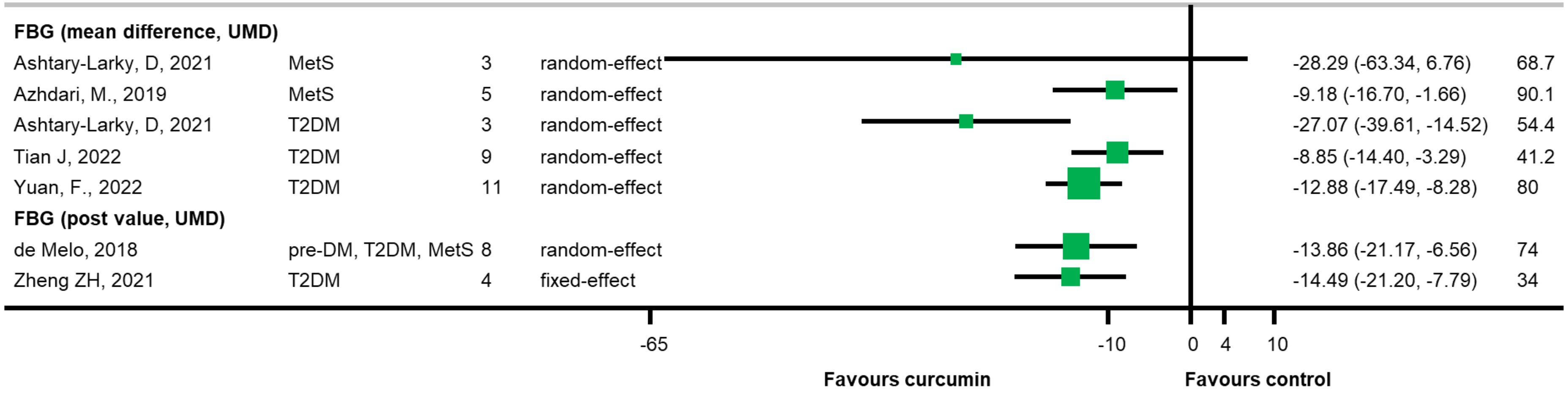

Supplement: S1 File — (ZIP) [file pone.0288997.s002.zip › Fig S18.pdf]
