# Supplementary material for: Effects of turmeric (Curcuma longa) supplementation on glucose metabolism in diabetes mellitus and metabolic syndrome: An umbrella review and updated meta-analysis
Source: PLoS One. 2023 Jul 20;18(7):e0288997. doi: 10.1371/journal.pone.0288997 (PMC10359013; doi:10.1371/journal.pone.0288997)

Fig S19. Forest plot of difference in changes of post-intervention value and change from baseline for hemoglobin A1C (HbA1C).

Study	Patients	Trial	Model	Mean difference (95% CI)	I^2
**HbA1c (mean difference, UMD)**					
Tian J, 2022	T2DM	9	random-effect	-0.54 (-0.81, -0.27)	65.2
Yuan, F., 2022	T2DM	10	random-effect	-0.41 (-0.56, -0.26)	73
Zhang, T, 2021	T2DM	5	fixed-effect	-0.70 (-0.87, -0.54)	0
**HbA1c (mean difference, SMD)**					
Altobelli E, 2021	T2DM	5	random-effect	-0.42 (-0.72, -0.11)	42.42
Huang, J, 2019	NAFLD/MetS	3	random-effect	-0.24 (-0.85, 0.37)	80.6
Huang, J, 2019	T2DM	5	random-effect	-0.46 (-0.71, -0.20)	44.9
Macena ML, 2022	T2DM	1	random-effect	0.96 (-0.28, 2.20)	-
Tabrizi R, 2018	T2DM	6	random-effect	-1.06 (-1.51, -0.60)	83
**HbA1c (post value, UMD)**					
de Melo, 2018	pre-DM, T2DM, MetS	7	random-effect	-0.54 (-1.09, -0.00)	90
Zheng ZH, 2021	T2DM	3	fixed-effect	-0.85 (-1.16, -0.54)	0

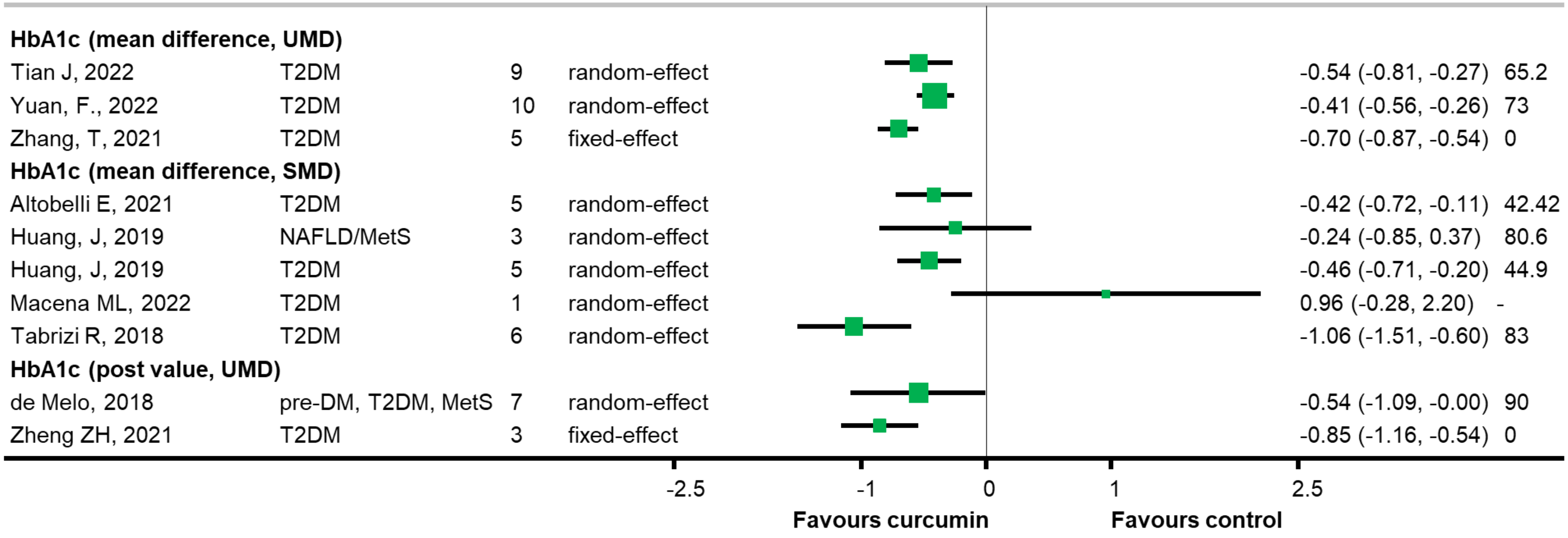

Supplement: S1 File — (ZIP) [file pone.0288997.s002.zip › Fig S19.pdf]
