# Supplementary material for: Effects of turmeric (Curcuma longa) supplementation on glucose metabolism in diabetes mellitus and metabolic syndrome: An umbrella review and updated meta-analysis
Source: PLoS One. 2023 Jul 20;18(7):e0288997. doi: 10.1371/journal.pone.0288997 (PMC10359013; doi:10.1371/journal.pone.0288997)

Fig S20. Forest plot of difference in changes of post-intervention value and change from baseline for homeostatic model assessment for insulin resistance (HOMA-IR).

Study	Patients	Trial	Model	Mean difference (95% CI)	I^2
**HOMA-IR (mean difference, UMD)**					
Zhang, T, 2021	T2DM	4	random-effect	-1.76 (-3.65, 0.13)	95
**HOMA-IR (mean difference, SMD)**					
Altobelli E, 2021	T2DM	4	random-effect	-0.41 (-0.66, -0.22)	0
Huang, J, 2019	NAFLD/MetS	3	random-effect	-0.28 (-0.55, -0.01)	0
Huang, J, 2019	T2DM	4	random-effect	-0.36 (-0.76, 0.04)	82
Tabrizi R, 2018	T2DM	5	random-effect	-0.80 (-1.58, -0.02)	95.3
**HOMA-IR (post value, UMD)**					
de Melo, 2018	pre-DM, T2DM, DLP	3	random-effect	-1.26 (-3.71, 1.19)	96

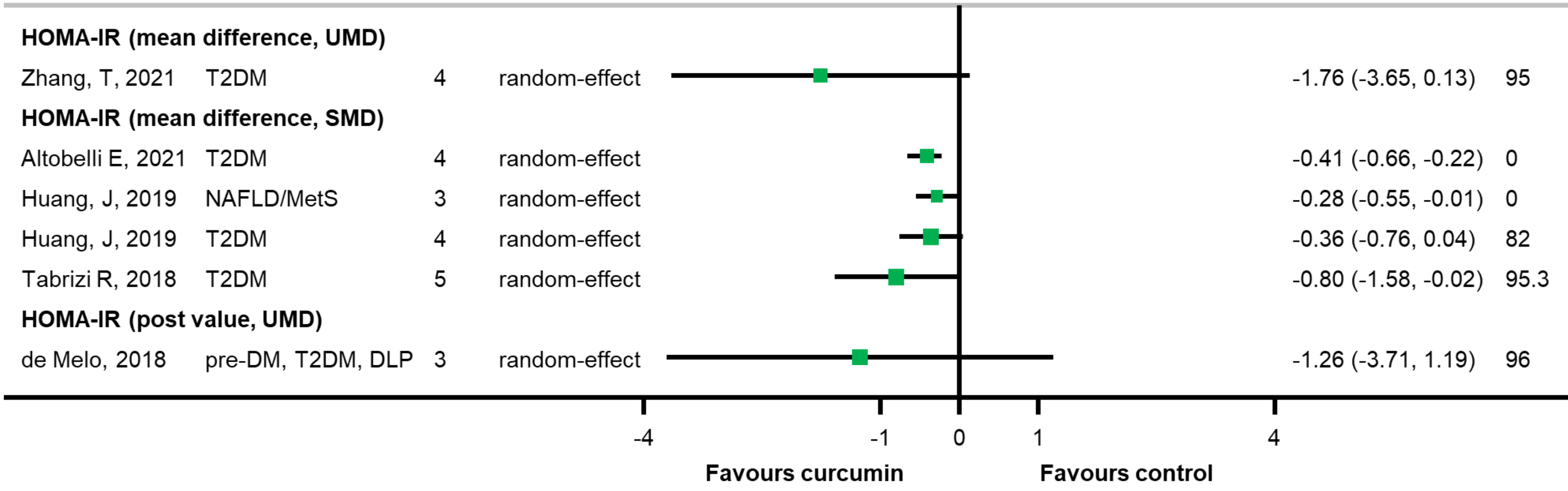

Supplement: S1 File — (ZIP) [file pone.0288997.s002.zip › Fig S20.pdf]
