# Supplementary material for: Effects of turmeric (Curcuma longa) supplementation on glucose metabolism in diabetes mellitus and metabolic syndrome: An umbrella review and updated meta-analysis
Source: PLoS One. 2023 Jul 20;18(7):e0288997. doi: 10.1371/journal.pone.0288997 (PMC10359013; doi:10.1371/journal.pone.0288997)

Fig S22. Forest plot of difference in changes of post-intervention value and change from baseline for total cholesterol (TC).

Study	Patients	Trial	model	Mean difference (95% CI)	I^2
**TC (mean difference, SMD)**					
Altobelli E, 2021	T2DM	5	random-effect	-0.30 (-0.53, -0.07)	0
Tabrizi R, 2018	T2DM	8	random-effect	-0.48 (-0.99, 0.02)	87.4

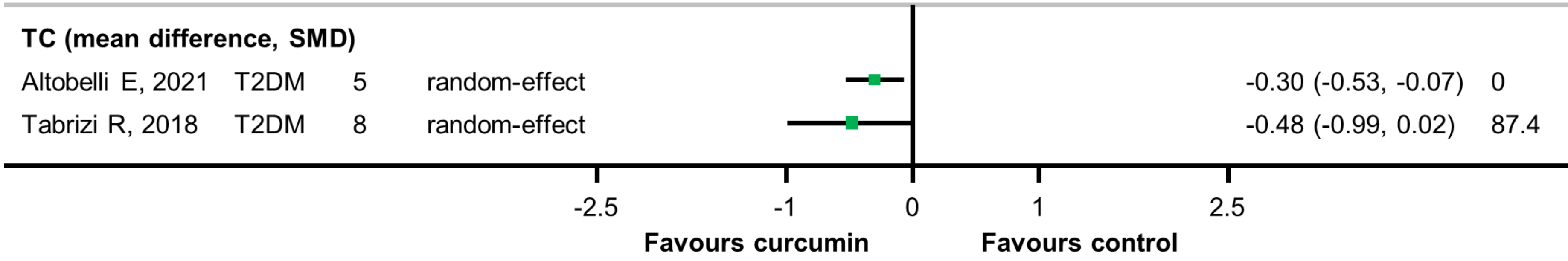

Study	Patients	Trial	Model	Mean difference (95% CI)	I^2
**TC (mean difference, UMD)**					
Ashtary-Larky, D, 2021	MetS	1	random-effect	10.40 (-11.09, 31.89)	-
Ashtary-Larky, D, 2021	T2DM	2	random-effect	-6.24 (-35.65, 23.17)	90.9
Tian J, 2022	T2DM	9	random-effect	-8.91 (-14.18, -3.63)	28.9
Yuan, F., 2019	T2DM	14	random-effect	-12.00 (-20.00, -4.04)	46
Zhang, T, 2021	T2DM	4	random-effect	-2.00 (-39.91, 35.91)	95
**TC (post value, UMD)**					
Zheng ZH, 2021	T2DM	5	fixed-effect	-11.30 (-20.69, -1.91)	20

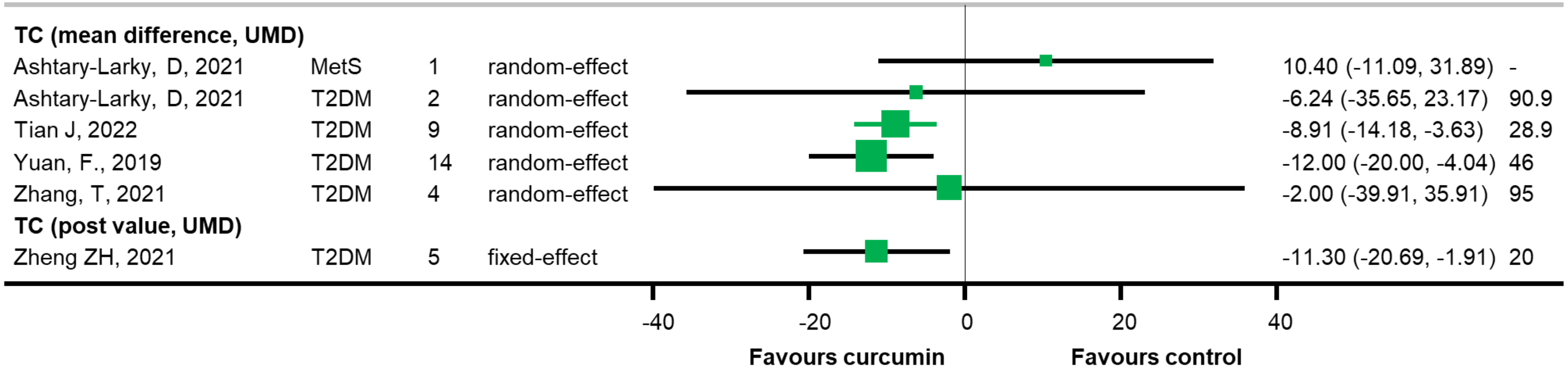

Supplement: S1 File — (ZIP) [file pone.0288997.s002.zip › Fig S22.pdf]
