# Supplementary material for: Effects of turmeric (Curcuma longa) supplementation on glucose metabolism in diabetes mellitus and metabolic syndrome: An umbrella review and updated meta-analysis
Source: PLoS One. 2023 Jul 20;18(7):e0288997. doi: 10.1371/journal.pone.0288997 (PMC10359013; doi:10.1371/journal.pone.0288997)

Fig S23. Forest plot of difference in changes of post-intervention value and change from baseline for triglyceride (TG).

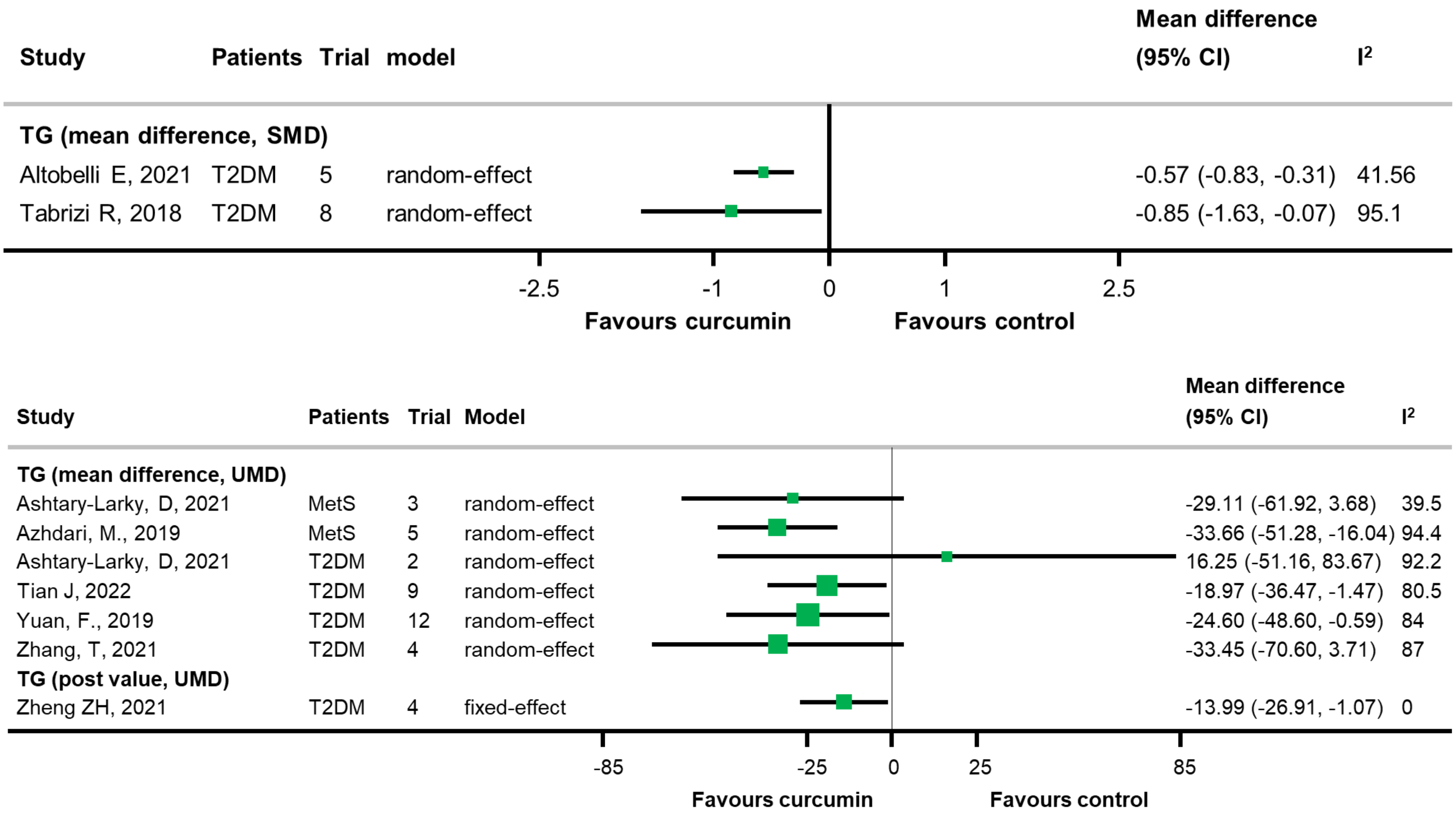

Supplement: S1 File — (ZIP) [file pone.0288997.s002.zip › Fig S23.pdf]
