# Supplementary material for: Effects of turmeric (Curcuma longa) supplementation on glucose metabolism in diabetes mellitus and metabolic syndrome: An umbrella review and updated meta-analysis
Source: PLoS One. 2023 Jul 20;18(7):e0288997. doi: 10.1371/journal.pone.0288997 (PMC10359013; doi:10.1371/journal.pone.0288997)

Fig S25. Forest plot of difference in changes of post-intervention value and change from baseline for high-density lipoprotein cholesterol (HDL-C).

Study	Patients	Trial	model	Mean difference (95% CI)	I^2
**HDL-C (mean difference, SMD)**					
Altobelli E, 2021	T2DM	5	random-effect	0.22 (-0.08, 0.52)	45.91
Tabrizi R, 2018	T2DM	8	random-effect	0.36 (-0.19, 0.90)	89.2

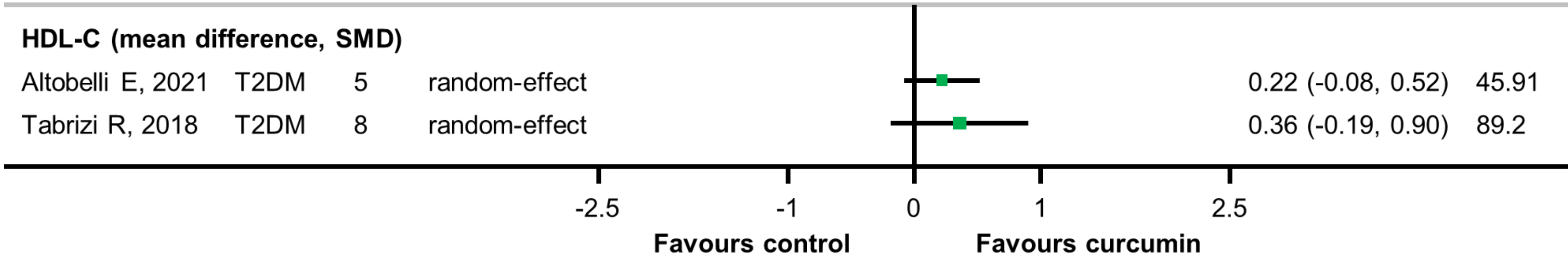

Study	Patients	Trial	Model	Mean difference (95% CI)	I^2
**HDL-C (mean difference, UMD)**					
Ashtary-Larky, D, 2021	MetS	3	random-effect	5.66 (3.34, 7.98)	0
Azhdari, M., 2019	MetS	5	random-effect	4.89 (4.59, 5.18)	98.6
Ashtary-Larky, D, 2021	T2DM	2	random-effect	6.84 (-2.85, 16.53)	93.4
Tian J, 2022	T2DM	9	random-effect	0.32 (-0.74, 1.37)	19.1
Yuan, F., 2019	T2DM	16	random-effect	2.74 (-0.02, 5.50)	77
Zhang, T, 2021	T2DM	5	random-effect	2.26 (-2.03, 6.55)	90
HDL-C (post value, UMD)					
Zheng ZH, 2021	T2DM	6	fixed-effect	2.92 (1.65, 4.19)	0

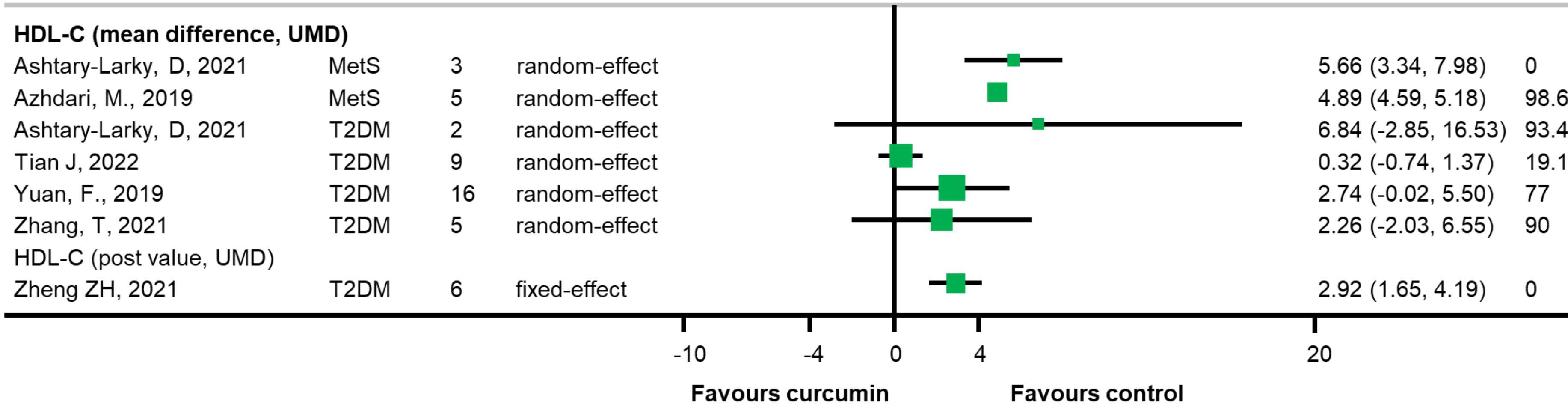

Supplement: S1 File — (ZIP) [file pone.0288997.s002.zip › Fig S25.pdf]
