# Supplementary material for: Effects of turmeric (Curcuma longa) supplementation on glucose metabolism in diabetes mellitus and metabolic syndrome: An umbrella review and updated meta-analysis
Source: PLoS One. 2023 Jul 20;18(7):e0288997. doi: 10.1371/journal.pone.0288997 (PMC10359013; doi:10.1371/journal.pone.0288997)

Fig S28. Forest plot of difference in changes of post-intervention value and change from baseline for C-reactive protein (CRP).

Study	Patients	Trial	Model	Mean difference (95% CI)	I^2
**CRP (mean difference, UMD)**					
Ashtary-Larky, D, 2021	MetS	2	random-effect	-0.64 (-1.52, 0.24)	86.2
Panahi Y, 2015	MetS	8	random-effect	-2.20 (-3.96, -0.44)	95
Ashtary-Larky, D, 2021	T2DM	1	random-effect	-3.60 (-8.27, 1.07)	-
**CRP (mean difference, SMD)**					
Macena ML, 2022	T2DM	1	random-effect	-1.60 (-3.14, -0.06)	-

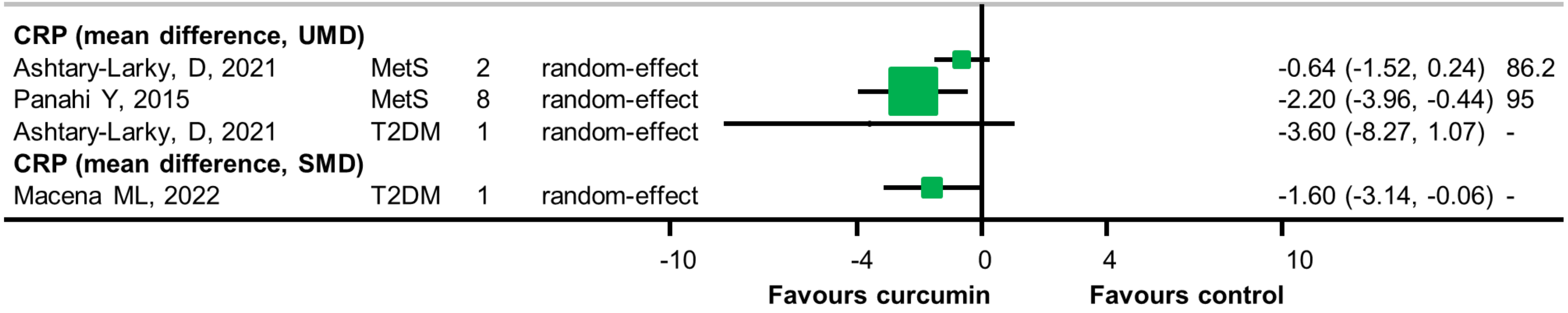

Supplement: S1 File — (ZIP) [file pone.0288997.s002.zip › Fig S28.pdf]
