# Supplementary material for: Effects of turmeric (Curcuma longa) supplementation on glucose metabolism in diabetes mellitus and metabolic syndrome: An umbrella review and updated meta-analysis
Source: PLoS One. 2023 Jul 20;18(7):e0288997. doi: 10.1371/journal.pone.0288997 (PMC10359013; doi:10.1371/journal.pone.0288997)

Fig S29. Forest plot of difference in changes of post-intervention value and change from baseline for high-sensitivity C-reactive protein (hs-CRP).

Study	Patients	Trial	Model	Mean difference (95% CI)	I^2
**hs-CRP (mean difference, SMD)**					
Gorabi AM, 2022	MetS	3	random-effect	-0.81 (-2.72, 1.10)	94.3
Gorabi AM, 2022	T2DM	4	random-effect	-0.17 (-1.75, 1.42)	65.8

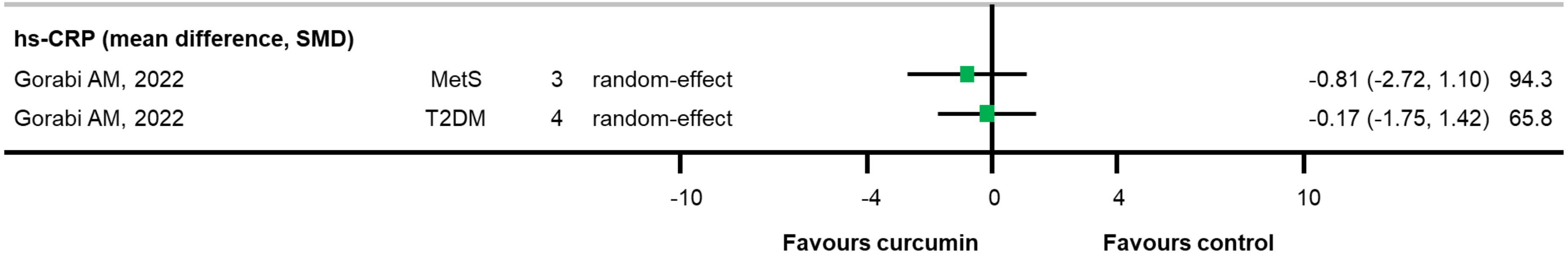

Supplement: S1 File — (ZIP) [file pone.0288997.s002.zip › Fig S29.pdf]
