# Supplementary material for: Effects of turmeric (Curcuma longa) supplementation on glucose metabolism in diabetes mellitus and metabolic syndrome: An umbrella review and updated meta-analysis
Source: PLoS One. 2023 Jul 20;18(7):e0288997. doi: 10.1371/journal.pone.0288997 (PMC10359013; doi:10.1371/journal.pone.0288997)
Supplement: S1 File — (ZIP) [file pone.0288997.s002.zip › Fig S30.pdf]

Fig S30. Funnel plots of the studies investigating the effects of *Curcuma longa* supplementation on fasting blood glucose (FBG).

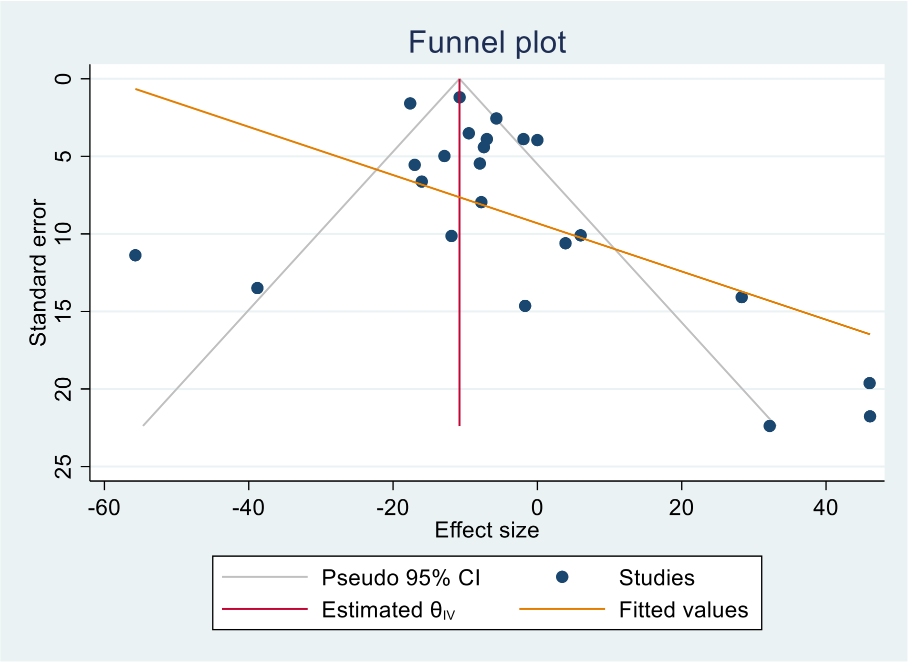

a. Post-intervention value

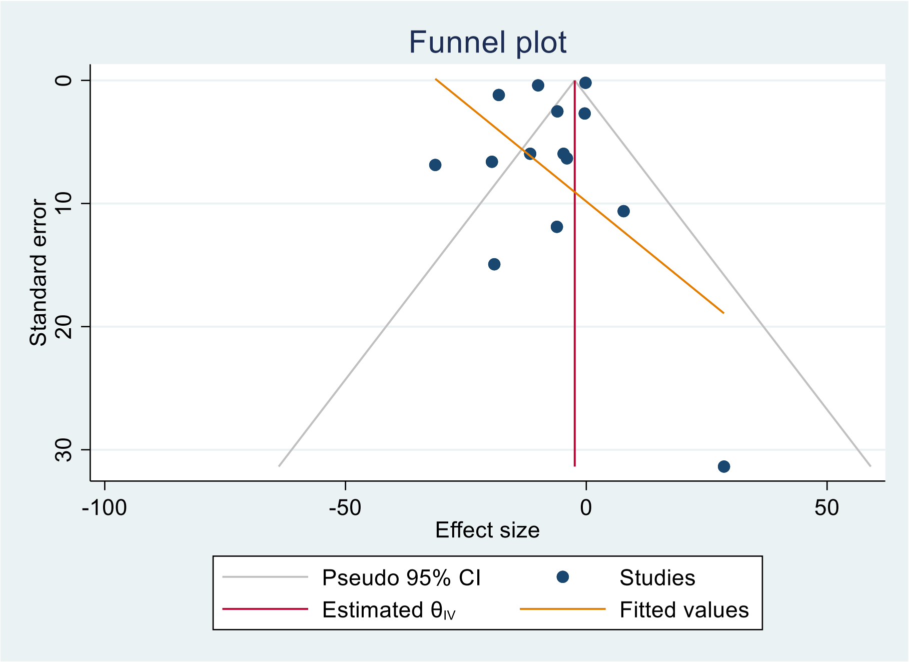

b. Change from baseline
