# Supplementary material for: Effects of turmeric (Curcuma longa) supplementation on glucose metabolism in diabetes mellitus and metabolic syndrome: An umbrella review and updated meta-analysis
Source: PLoS One. 2023 Jul 20;18(7):e0288997. doi: 10.1371/journal.pone.0288997 (PMC10359013; doi:10.1371/journal.pone.0288997)
Supplement: S1 File — (ZIP) [file pone.0288997.s002.zip › Table S1.pdf]

Table S1. Search algorithms.

Database	Step	Search algorithm	Items found
**MEDLINE (Pubmed)**	#1	curcuma longa[MeSH Terms]	2,357
	#2	curcuma domestica[MeSH Terms]	2,357
	#3	curcumin[MeSH Terms]	13,093
	#4	curcuminoid[MeSH Terms]	13,563
	#5	turmeric[MeSH Terms]	2,357
	#6	tumeric[MeSH Terms]	14,580
	#7	#1 OR #1 OR #2 OR #3 OR #4 OR #5 OR #6	14,999
	#8	diabetes[MeSH Terms]	483,182
	#9	type 2 diabetes mellitus[MeSH Terms]	155,924
	#10	prediabetes[MeSH Terms]	8,189
	#11	dysglycemia	1,310
	#12	metabolic syndrome[MeSH Terms]	35,949
	#13	#8 OR #9 OR #10 OR #11 OR #12	512,361
	#14	glucose[MeSH Terms]	323,672
	#15	fasting plasma glucose[MeSH Terms]	17,795
	#16	glycated hemoglobin[MeSH Terms]	39,696
	#17	lipid[MeSH Terms]	1,235,148
	#18	homeostatic model assessment of insulin resistance[MeSH Terms]	9,920
	#19	HOMO-IR	4
	#20	blood pressure[MeSH Terms]	322,044
	#21	uric acid[MeSH Terms]	26,664
	#22	c-reactive protein[MeSH Terms]	51,015
	#23	interleukin 6[MeSH Terms]	69,821
	#24	#14 OR #15 OR #16 OR #17 OR #18 OR #19 OR #20 OR #21 #22 OR #23	1,909,998
	#25	#7 AND #13 AND #24	198
	#26	Meta-analysis: Update search from 2021 to 2022	1
	#27	Individual work: Update search from 2021 to 2022	92
**EMBASE**	#1	'curcuma longa'	6,576
	#2	'curcuma domestica'	95
	#3	curcumin*	32,980
	#4	curcuminoid*	1,884
	#5	turmeric	5,832
	#6	tumeric	121
	#7	#1 OR #1 OR #2 OR #3 OR #4 OR #5 OR #6	37,360
	#8	diabetes	1,354,405
	#9	'type 2 diabetes mellitus'	80,849
	#10	prediabetes	16,964
	#11	'metabolic syndrome'	120,544
	#12	dysglycemia	2,958
	#13	#8 OR #9 OR #10 OR #11 OR #12	1,412,375
	#14	glucose	971,805
	#15	'fasting plasma glucose'	21,344
	#16	'glycated hemoglobin'	14,003
	#17	lipid	872,536
	#18	'homeostatic model assessment of insulin resistance'	1,758
	#19	'homo ir'	23
	#20	'blood pressure'	709,506
	#21	'uric acid'	68,441
	#22	'c-reactive protein'	230,845
	#23	'interleukin-6'	304,956
	#24	#14 OR #15 OR #16 OR #17 OR #18 OR #19 OR #20 OR #21 #22 OR #23	2,765,979
	#25	#7 AND #13 AND #24	1,391
	#26	Meta-analysis: Update search from 2021 to 2022	1
	#27	Individual work: Update search from 2021 to 2022	232
	#1	..nlp curcuma longa	751

Database	Step	Search algorithm	Items found
**CENTRAL via OVID**	#2	..nlp curcuma domestica	477
	#3	..nlp curcumin	1,440
	#4	..nlp curcuminoid	222
	#5	..nlp turmeric	768
	#6	..nlp tumeric	1,721
	#7	#1 OR #1 OR #2 OR #3 OR #4 OR #5 OR #6	1,556
	#8	..nlp diabetes	14,173
	#9	..nlp type 2 diabetes mellitus	15,083
	#10	..nlp prediabetes	5,020
	#11	..nlp dysglycemia	264
	#12	..nlp metabolic syndrome	7,780
	#13	#8 OR #9 OR #10 OR #11 OR #12	102,319
	#14	..nlp glucose	12,462
	#15	..nlp fasting plasma glucose	10,333
	#16	..nlp glycated hemoglobin	7,309
	#17	..nlp lipid	10,367
	#18	..nlp homeostatic model assessment of insulin resistance	10,150
	#19	..nlp HOMO-IR	0
	#20	..nlp blood pressure	12,387
	#21	..nlp uric acid	4,717
	#22	..nlp c-reactive protein	27,397
	#23	..nlp interleukin-6	17,570
	#24	#14 OR #15 OR #16 OR #17 OR #18 OR #19 OR #20 OR #21 #22 OR #23	207,289
	#25	#7 AND #13 AND #24	151
	#26	Meta-analysis: Update search from 2021 to 2022	0
	#27	Individual work: Update search from 2021 to 2022	24
**Scopus**	#1	ALL (curcuma)	41,259
	#2	ALL ("curcuma longa")	29,709
	#3	ALL ("curcuma domestica")	1,217
	#4	ALL (curcumin)	188,475
	#5	ALL (curcuminoid)	9,716
	#6	ALL (turmeric)	32,101
	#7	ALL (tumeric)	1,230
	#8	#1 OR #1 OR #2 OR #3 OR #4 OR #5 OR #6 OR #7	220,070
	#9	ALL (diabetes) OR ALL ("diabetes mellitus")	2,437,705
	#10	ALL ("type 2") OR ALL ("type II")	1,884,828
	#11	#9 AND #10	922,678
	#12	ALL ("prediabetes")	44,054
	#13	ALL ("dysglycemia")	9,274
	#14	ALL ("metabolic syndrome")	450,957
	#15	#11 OR #12 OR #13 OR #14	1,040,208
	#16	ALL (glucose)	2,185,314
	#17	ALL ("fasting plasma glucose")	53,036
	#18	ALL ("glycated hemoglobin")	42,081
	#19	ALL ("lipid")	2,802,937
	#20	ALL ("insulin resistance")	494,065
	#21	ALL ("HOMO-IR")	9
	#22	ALL ("blood pressure")	1,136,257
	#23	ALL ("uric acid")	141,794
	#24	ALL ("c-reactive protein")	347,502
	#25	ALL ("interleukin 6")	506,121
	#26	#16 OR #17 OR #18 OR #19 OR #20 OR #21 OR #22 OR #23 OR #24 OR #25	5,836,425
	#27	#8 AND #15 AND #26	20,367

Database	Step	Search algorithm	Items found
	#28	Limited (Medicine, Human)<br>Excluded (Editorial, Short survey, Note, Book chapter, Letter, Retracted, Book, Book series, Mathematics, Computer Science, Physics and Astronomy, Veterinary, Dentistry, Social Sciences, Materials Science, Chemical Engineering, Environmental Science, Engineering, Chemistry, Health Professions, Psychology, Agricultural and Biological Sciences, Neuroscience, Immunology and Microbiology, Nursing, Biochemistry, Genetics and Molecular Biology)	1,748
	#29	Meta-analysis: Update search from 2021 to 2022	2
	#30	Individual work: Update search from 2021 to 2022	426
