# Supplementary material for: Effects of turmeric (Curcuma longa) supplementation on glucose metabolism in diabetes mellitus and metabolic syndrome: An umbrella review and updated meta-analysis
Source: PLoS One. 2023 Jul 20;18(7):e0288997. doi: 10.1371/journal.pone.0288997 (PMC10359013; doi:10.1371/journal.pone.0288997)
Supplement: S1 File — (ZIP) [file pone.0288997.s002.zip › Table S10.pdf]

Table S10. Subgroup analysis of difference in change of HbA1C (%) within 4 months between *Curcuma longa* supplementation and control group.

Outcomes	Post-intervention value						Change from baseline					
	Trials (n)	Control (n)	Curcumin (n)	Mean difference (95% CI)	P value	Heterogeneity (I^2)	Trials (n)	Control (n)	Curcumin (n)	Mean difference (95% CI)	P value	Heterogeneity (I^2)
Baseline BMI												
• < 30 kg/m2	18	775	794	–0.128 (–0.339, 0.083)	0.235	83.8%	9	330	342	**–0.488 (–0.709, –0.268)**	**<0.001**	67.9%
• ≥ 30 kg/m^2	2	61	82	–0.508 (–1.340, 0.323)	0.231	77.3%	2	59	82	**–0.705 (–1.024, –0.387)**	**<0.001**	0.0%
Baseline TC												
• < 200 mg/dL	9	347	368	–0.176 (–0.374, 0.023)	0.082	53.8%	4	138	137	**–0.638 (–1.188, –0.087)**	**0.023**	50.3%
• ≥ 200 mg/dL	6	249	272	–0.075 (–0.725, 0.575)	0.821	90.5%	4	145	184	**–0.546 (–0.824, –0.268)**	**<0.001**	60.3%
Baseline TG												
• < 150 mg/dL	5	140	156	–0.170 (–0.439, 0.099)	0.215	82.2%	2	60	81	–0.928 (–1.924, 0.068)	0.068	41.6%
• ≥ 150 mg/dL	13	572	597	0.049 (–0.314, 0.412)	0.791	85.9%	7	261	278	–0.417 (–0.679, –0.155)	0.002	65.5%
Baseline LDL-C												
• < 100 mg/dL	5	196	194	–0.157 (–0.960, 0.645)	0.701	80.7%	3	106	104	–0.672 (–1.285, –0.059)	0.032	64.5%
• ≥ 100 mg/dL	12	493	531	–0.088 (–0.322, 0.147)	0.463	87.9%	6	215	255	–0.409 (–0.717, –0.102)	0.009	69.5%
Baseline HDL-C												
• < 45 mg/dL	10	364	383	–0.085 (–0.451, 0.281)	0.649	85.0%	6	223	240	**–0.531 (–0.754, –0.307)**	**<0.001**	45.5%
• ≥ 45 mg/dL	7	325	342	–0.073 (–0.373, 0.227)	0.633	85.8%	3	98	119	–0.512 (–1.474, 0.449)	0.297	82.6%
Baseline FBG												
• < 130 mg/dL	6	240	266	–0.103 (–0.218, 0.011)	0.077	60.0%	2	51	75	–0.074 (–1.348, 1.201)	0.909	0.0%

Outcomes	Post-intervention value						Change from baseline					
	**Trials (n)**	**Control (n)**	**Curcumin (n)**	**Mean difference (95% CI)**	**P value**	**Heterogeneity (I^2)**	**Trials (n)**	**Control (n)**	**Curcumin (n)**	**Mean difference (95% CI)**	**P value**	**Heterogeneity (I^2)**
• ≥ 130 mg/dL	15	656	670	–0.107 (–0.468, 0.255)	0.562	85.7%	9	338	349	**–0.525 (–0.723, –0.328)**	**<0.001**	68.2%
Baseline HbA1C												
• < 7%	6	285	307	–0.060 (–0.208, 0.088)	0.425	76.5%	4	149	174	–0.241 (–0.566, 0.084)	0.146	0.0%
• ≥ 7%	15	611	629	–0.124 (–0.475, 0.227)	0.490	83.3%	7	240	250	**–0.583 (–0.793, –0.374)**	**<0.001**	68.5%
Baseline SBP												
• < 130 mmHg	7	243	264	–0.026 (–0.182, 0.130)	0.740	52.9%	2	53	50	**–0.381 (–0.615, –0.148)**	**0.001**	0.0%
• ≥ 130 mmHg	5	221	234	0.185 (–0.688, 1.058)	0.678	92.5%	3	107	119	–0.301 (–0.812, 0.210)	0.249	87.0%
Baseline DBP												
• < 90 mmHg	6	235	254	0.002 (–0.214, 0.218)	0.986	71.4%	2	66	63	–0.254 (–0.609, 0.100)	0.160	56.4%
• ≥ 90 mmHg	6	229	244	0.003 (–0.626, 0.631)	0.994	90.2%	3	94	106	–0.332 (–0.638, –0.027)	0.136	76.1%
Trial included patient												
• with DM	16	644	659	–0.130 (–0.432, 0.172)	0.399	85.7%	7	255	265	–0.594 (–0.791, –0.398)	**<0.001**	66.9%
• without DM	5	252	277	–0.098 (–0.253, 0.056)	0.212	67.3%	4	134	159	–0.093 (–0.468, 0.282)	0.627	0.0%
• with pre-DM	3	155	175	–0.091 (–0.221, 0.038)	0.165	79.8%	1	19	42	–0.100 (–1.709, 1.509)	0.903	–
• without pre-DM	18	741	761	–0.103 (–0.411, 0.204)	0.511	83.1%	10	370	382	–0.522 (–0.716, –0.328)	**<0.001**	64.7%
• with MetS	4	126	139	–0.169 (–0.833, 0.495)	0.618	90.3%	4	126	139	–0.369 (–0.828, 0.089)	0.114	65.6%
• without MetS	17	770	797	–0.116 (–0.290, 0.058)	0.191	79.2%	7	263	285	**–0.559 (–0.812, –0.306)**	**<0.001**	61.5%
**CL preparations**												

Outcomes	Post-intervention value						Change from baseline					
	**Trials (n)**	**Control (n)**	**Curcumin (n)**	**Mean difference (95% CI)**	**P value**	**Heterogeneity (I^2)**	**Trials (n)**	**Control (n)**	**Curcumin (n)**	**Mean difference (95% CI)**	**P value**	**Heterogeneity (I^2)**
• Whole preparation	4	104	120	–0.134 (–0.304, 0.037)	0.235	74.8%	2	51	62	**–0.686 (–0.793, –0.580)**	**<0.001**	0.0%
○ Dose < 1,500 mg/day				NA						NA		
○ Dose ≥ 1,500 mg/day	4	104	120	–0.301 (–0.797, 0.195)	0.235	74.8%	2	51	62	**–0.686 (–0.793, –0.580)**	**<0.001**	0.0%
• Extract preparation	9	477	500	–0.077 (–0.237, 0.084)	0.348	65.0%	3	98	96	**–0.278 (–0.535, –0.021)**	0.034	15.3%
○ Dose < 1,000 mg/day	4	134	158	0.041 (–0.480, 0.563)	0.877	78.8%	1	38	38	–0.030 (–0.443, 0.383)	0.887	–
○ Dose ≥ 1,000 mg/day	5	343	342	–0.088 (–0.326, 0.151)	0.472	53.9%	2	60	58	**–0.395 (–0.636, –0.154)**	**0.001**	0.0%
• Bioavailability-enhanced preparation	8	315	316	–0.176 (–0.668, 0.317)	0.484	88.4%	6	240	266	**–0.591 (–0.921, –0.261)**	**<0.001**	60.1%

Abbreviations: BMI, body mass index; CL, *Curcuma longa*; DBP, diastolic blood pressure; DM, diabetic mellitus; FBG, fasting blood glucose; HbA1C, hemoglobin A1C; HDL-c, high-density lipoprotein cholesterol; LDL-c, low-density lipoprotein cholesterol; MetS, metabolic syndrome; NA, not applicable; pre-DM, pre-diabetic mellitus; SBP, systolic blood pressure; TC, total cholesterol; TG, triglyceride.
