# Supplementary material for: Effects of turmeric (Curcuma longa) supplementation on glucose metabolism in diabetes mellitus and metabolic syndrome: An umbrella review and updated meta-analysis
Source: PLoS One. 2023 Jul 20;18(7):e0288997. doi: 10.1371/journal.pone.0288997 (PMC10359013; doi:10.1371/journal.pone.0288997)
Supplement: S1 File — (ZIP) [file pone.0288997.s002.zip › Table S11.pdf]

**Table S11. Post-hoc analysis of difference in change of secondary outcomes within 4 months stratified by *Curcuma longa* preparations between *C. longa* supplementation and control group.**

| Outcomes | Post-intervention value | | | | | | Change from baseline | | | | | |
|---|---|---|---|---|---|---|---|---|---|---|---|---|
| | Trials (n) | Control (n) | Curcumin (n) | Mean difference (95% CI) | P value | Heterogeneity ($I^2$) | Trials (n) | Control (n) | Curcumin (n) | Mean difference (95% CI) | P value | Heterogeneity ($I^2$) |
| **HOMA-IR** | | | | | | | | | | | | |
| • Whole preparation | 3 | 81 | 92 | **–0.545 (–0.627, –0.463)** | **<0.001** | 0.0% | 2 | 51 | 62 | **–0.546 (–0.628, –0.465)** | **<0.001** | 0.0% |
| • Extract preparation | 5 | 343 | 342 | –0.695 (–1.571, 0.180) | 0.120 | 78.1% | 2 | 66 | 63 | **–0.634 (–1.083, –0.185)** | **0.006** | 0.0% |
| • Bioavailability-enhanced preparation | 4 | 158 | 163 | 1.523 (–1.154, 4.201) | 0.265 | 95.4% | 2 | 82 | 86 | –0.108 (–0.234, 0.019) | 0.095 | 0.0% |
| **Insulin** | | | | | | | | | | | | |
| • Whole preparation | 3 | 81 | 92 | **–0.704 (–0.837, –0.572)** | **0.679** | 0.0% | 2 | 51 | 62 | **–0.700 (–0.914, –0.486)** | **<0.001** | 0.0% |
| • Extract preparation | 5 | 310 | 329 | –0.358 (–1.027, 0.310) | 0.293 | 58.9% | 2 | 66 | 63 | –0.723 (–2.157, 0.712) | 0.323 | 26.8% |
| • Bioavailability-enhanced preparation | 4 | 158 | 163 | –0.449 (–2.579, 1.680) | 0.679 | 75.1% | 2 | 82 | 86 | –0.432 (–1.494, 0.631) | 0.426 | 11.5% |
| **BMI** | | | | | | | | | | | | |
| • Whole preparation | 4 | 137 | 153 | –0.587 (–2.543, 1.369) | 0.557 | 81.0% | 3 | 91 | 102 | –1.134 (–2.330, 0.061) | 0.063 | 97.3% |
| • Extract preparation | 6 | 280 | 299 | 0.386 (–1.046, 1.819) | 0.597 | 79.0% | 5 | 152 | 171 | –0.259 (–0.731, 0.212) | 0.281 | 88.8% |
| • Bioavailability-enhanced preparation | 7 | 257 | 257 | –0.768 (–1.715, 0.179) | 0.112 | 55.1% | 5 | 203 | 205 | **–0.435 (–0.837, –0.034)** | **0.033** | 85.4% |
| **TC** | | | | | | | | | | | | |
| • Whole preparation | 5 | 164 | 175 | **–12.744 (–24.567, –0.922)** | **0.035** | 81.5% | 4 | 111 | 122 | –6.599 (34.310, 21.112) | 0.641 | 98.9% |
| • Extract preparation | 6 | 263 | 284 | **–8.234 (–14.313, –2.156)** | **0.008** | 0.0% | 4 | 114 | 133 | **–6.669 (–11.317, –2.021)** | **0.005** | 18.2% |
| • Bioavailability-enhanced preparation | 6 | 231 | 234 | 12.547 (–21.027, 46.120) | 0.464 | 95.2% | 6 | 215 | 237 | –1.720 (–9.662, 6.222) | 0.671 | 98.8% |

| Outcomes | Post-intervention value | | | | | | Change from baseline | | | | | |
|---|---|---|---|---|---|---|---|---|---|---|---|---|
| | **Trials (n)** | **Control (n)** | **Curcumin (n)** | **Mean difference (95% CI)** | **P value** | **Heterogeneity ($I^2$)** | **Trials (n)** | **Control (n)** | **Curcumin (n)** | **Mean difference (95% CI)** | **P value** | **Heterogeneity ($I^2$)** |
| **TG** | | | | | | | | | | | | |
| • Whole preparation | 6 | 187 | 203 | **–12.463 (–23.225, –1.701)** | **0.023** | 62.0% | 4 | 111 | 122 | –22.025 (–50.338, 6.288) | 0.127 | 87.7% |
| • Extract preparation | 8 | 361 | 382 | –10.402 (–25.596, 4.792) | 0.180 | 72.6% | 5 | 152 | 171 | –12.953 (–27.615, 1.710) | 0.083 | 54.6% |
| • Bioavailability-enhanced preparation | 7 | 249 | 249 | **13.983 (3.471, 24.496)** | **0.009** | 6.8% | 5 | 196 | 195 | –5.511 (–20.318, 9.297) | 0.466 | 77.1% |
| **LDL** | | | | | | | | | | | | |
| • Whole preparation | 5 | 164 | 175 | –9.995 (–22.903, 2.912) | 0.129 | 93.0% | 4 | 111 | 122 | –1.301 (–14.266, 11.664) | 0.844 | 86.1% |
| • Extract preparation | 8 | 361 | 382 | **–7.273 (–11.962, –2.585)** | **0.002** | 0.0% | 5 | 152 | 171 | **–8.810 (–15.433, –2.186)** | **0.009** | 57.4% |
| • Bioavailability-enhanced preparation | 7 | 249 | 249 | –0.829 (–7.292, 5.634) | 0.802 | 39.9% | 6 | 215 | 237 | –2.887 (–10.722, 4.949) | 0.470 | 95.8% |
| **HDL** | | | | | | | | | | | | |
| • Whole preparation | 5 | 164 | 175 | 2.608 (–2.471, 7.687) | 0.314 | 92.6% | 4 | 111 | 122 | 3.742 (–1.593, 9.077) | 0.169 | 85.3% |
| • Extract preparation | 8 | 361 | 382 | 2.168 (–0.696, 5.032) | 0.138 | 81.9% | 5 | 152 | 171 | 1.808 (–3.651, 7.266) | 0.516 | 95.1% |
| • Bioavailability-enhanced preparation | 7 | 249 | 249 | **3.000 (1.374, 4.627)** | **<0.001** | 13.0% | 6 | 215 | 237 | **1.566 (0.162, 2.969)** | **0.029** | 87.6% |
| **SBP** | | | | | | | | | | | | |
| • Whole preparation | 4 | 117 | 133 | –1.074 (–7.435, 5.288) | 0.741 | 80.2% | 3 | 71 | 82 | –0.863 (–11.349, 9.624) | 0.872 | 89.5% |
| • Extract preparation | 3 | 173 | 169 | 0.016 (–0.854, 0.885) | 0.972 | 0.0% | 2 | 66 | 63 | 0.053 (–0.769, 0.876) | 0.899 | 0.0% |
| • Bioavailability-enhanced preparation | 3 | 101 | 99 | –4.097 (–13.581, 5.387) | 0.397 | 66.7% | 2 | 98 | 99 | 0.596 (–3.706, 4.898) | 0.786 | 16.4% |
| **DBP** | | | | | | | | | | | | |
| • Whole preparation | 4 | 117 | 133 | **–3.605 (–6.830, –0.380)** | **0.028** | 75.5% | 3 | 71 | 82 | –2.226 (–8.257, 3.805) | 0.469 | 75.9% |

| Outcomes | Post-intervention value | | | | | | Change from baseline | | | | | |
|---|---|---|---|---|---|---|---|---|---|---|---|---|
| | **Trials (n)** | **Control (n)** | **Curcumin (n)** | **Mean difference (95% CI)** | **P value** | **Heterogeneity ($I^2$)** | **Trials (n)** | **Control (n)** | **Curcumin (n)** | **Mean difference (95% CI)** | **P value** | **Heterogeneity ($I^2$)** |
| • Extract preparation | 2 | 145 | 144 | 0.202 (–2.818, 3.222) | 0.896 | 15.4% | 2 | 66 | 63 | –0.860 (–3.875, 2.155) | 0.576 | 86.0% |
| • Bioavailability-enhanced preparation | 3 | 101 | 99 | **–4.099 (–6.252, –1.946)** | **<0.001** | 0.0% | 2 | 98 | 99 | –1.181 (–6.707, 4.345) | 0.675 | 75.4% |
| **CRP** | | | | | | | | | | | | |
| • Whole preparation | 1 | 63 | 63 | NA | | | 1 | 19 | 42 | NA | | |
| • Extract preparation | | | | NA | | | | | | NA | | |
| • Bioavailability-enhanced preparation | | | | NA | | | | | | NA | | |
| **hs-CRP** | | | | | | | | | | | | |
| • Whole preparation | 3 | 81 | 92 | –0.441 (–1.891, 1.010) | 0.552 | 73.2% | 3 | 91 | 102 | –0.224 (–0.440, –0.008) | 0.042 | 10.4% |
| • Extract preparation | 1 | 28 | 25 | –0.500 (–2.427, 1.427) | 0.611 | – | 1 | 28 | 25 | **–3.300 (–5.361, –1.239)** | **0.002** | – |
| • Bioavailability-enhanced preparation | 6 | 220 | 222 | –1.493 (–3.213, 0.227) | 0.089 | 94.6% | 4 | 180 | 185 | –0.568 (–1.776, 0.640) | 0.357 | 93.1% |
| **Uric acid** | | | | | | | | | | | | |
| • Whole preparation | | | | NA | | | | | | NA | | |
| • Extract preparation | 1 | 107 | 106 | NA | | | | | | NA | | |
| • Bioavailability-enhanced preparation | 1 | 18 | 15 | NA | | | | | | NA | | |
| **IL-6** | | | | | | | | | | | | |
| • Whole preparation | | | | NA | | | | | | NA | | |
| • Extract preparation | | | | NA | | | | | | NA | | |
| • Bioavailability-enhanced preparation | | | | NA | | | | | | NA | | |

**Abbreviations:** BMI, body mass index; CRP, C-reactive protein; DBP, diastolic blood pressure; FBG, fasting blood glucose; HbA1C, hemoglobin A1C; HDL-C, high-density lipoprotein cholesterol; HOMA-IR, homeostatic model assessment for insulin resistance; hs-CRP, high sensitivity C-reactive protein; IL-6, interleukin 6; LDL-C, low-density lipoprotein cholesterol; NA, not applicable; SBP, systolic blood pressure; SCr, serum creatinine; TC, total cholesterol; TG, triglyceride.
