# Supplementary material for: Effects of turmeric (Curcuma longa) supplementation on glucose metabolism in diabetes mellitus and metabolic syndrome: An umbrella review and updated meta-analysis
Source: PLoS One. 2023 Jul 20;18(7):e0288997. doi: 10.1371/journal.pone.0288997 (PMC10359013; doi:10.1371/journal.pone.0288997)
Supplement: S1 File — (ZIP) [file pone.0288997.s002.zip › Table S12.pdf]

Table S12. Sensitivity analysis of primary outcomes.

Outcomes	Post-intervention value				Change from baseline			
	Main analyses	Fixed effect model	Excluded small sample size study (<25th percentiles)	Excluded high risk of bias studies	Main analyses	Fixed effect model	Excluded small sample size study (<25th percentiles)	Excluded high risk of bias studies
**FBG (mg/dL)**								
• MD (95% CI)	**–8.129 (–12.175, –4.084)**	**–10.790 (–12.257, –9.323)**	**–8.485 (–13.088, –3.881)**	**–5.795 (–10.411, –1.179)**	**–8.833 (–13.907, –3.758**	**–2.394 (–2.730, –2.059)**	**–7.828 (–12.954, –2.702)**	**–9.766 (–14.736, –4.796)**
• I^2	89.2%	82.9%	87.4%	90.5%	99.6%	98.4%	99.6%	96.2%
• Number of studies	23	23	18	18	14	14	11	10
**HbA1C (%)**								
• MD (95% CI)	–0.134 (–0.304, 0.037)	**–0.151 (–0.201, –0.101)**	–0.141 (–0.332, 0.051)	–0.117 (–0.295, 0.061)	**–0.517 (–0.707, –0.327**	**–0.611 (–0.695, –0.527)**	**–0.492 (–0.731, –0.254)**	**–0.484 (–0.697, –0.270)**
• I^2	93.3%	87.6%	93.2%	92.7%	83.9%	78.4%	82.5%	85.6%
• Number of studies	21	21	16	16	11	11	9	7

Abbreviations: FBG, fasting blood glucose; HbA1C, hemoglobin A1C; MD, mean difference; MetS, metabolic syndrome; NA.
