# Supplementary material for: Effects of turmeric (Curcuma longa) supplementation on glucose metabolism in diabetes mellitus and metabolic syndrome: An umbrella review and updated meta-analysis
Source: PLoS One. 2023 Jul 20;18(7):e0288997. doi: 10.1371/journal.pone.0288997 (PMC10359013; doi:10.1371/journal.pone.0288997)
Supplement: S1 File — (ZIP) [file pone.0288997.s002.zip › Table S13.pdf]

Table S13. Assessment of small study effects by Egger's test for each outcome.

Outcomes	Post-intervention value		Change from baseline	
	Trials (n)	P value	Trials (n)	P value
FBG (mg/dL)	23	**0.0212**	14	0.8134
HbA1C (%)	21	0.2918	11	0.3867
HOMA-IR (%)	12	0.5680	6	0.5156
Insulin (μIU/mL)	12	0.1951	6	0.9647
BMI (kg/m^2)	17	**0.0003**	12	0.6639
TC (mg/dL)	17	0.1621	13	0.9755
TG (mg/dL)	21	0.5780	13	0.0625
LDL-C (mg/dL)	20	0.1137	14	0.1825
HDL-C (mg/dL)	20	0.6221	14	0.5834
SBP (mm Hg)	10	0.8864	6	0.0543
DBP (mm Hg)	9	0.3279	6	0.5994
CRP (mg/L)	1	NA	1	NA
hs-CRP (mg/L)	10	**0.0003**	7	0.2372
uric acid (mg/dL)	2	NA	NA	NA
IL-6 (pg/mL)	NA	NA	NA	NA

Abbreviations: BMI, body mass index; CRP, C-reactive protein; DBP, diastolic blood pressure; FBG, Fasting blood glucose; HbA1C, Hemoglobin A1C; HDL-c, High-density lipoprotein cholesterol; hs-CRP, High sensitivivity C-reactive protein; LDL-c, Low-density lipoprotein cholesterol; MetS, metabolic syndrome; NA, Not applicable; pre-DM, pre-diabetic mellitus; SBP, systolic blood pressure; TC, Total cholesterol; TG, Triglyceride.
