# Supplementary material for: Effects of turmeric (Curcuma longa) supplementation on glucose metabolism in diabetes mellitus and metabolic syndrome: An umbrella review and updated meta-analysis
Source: PLoS One. 2023 Jul 20;18(7):e0288997. doi: 10.1371/journal.pone.0288997 (PMC10359013; doi:10.1371/journal.pone.0288997)
Supplement: S1 File — (ZIP) [file pone.0288997.s002.zip › Table S14.pdf]

Table S14. Estimates of effects and quality ratings for comparison of treatment options in primary outcomes.

Certainty assessment							№ of patients		Effect		Certainty	Importance
№ of studies	Study design	Risk of bias	Inconsistency	Indirectness	Imprecision	Other considerations	Curcumin	Control	Relative (95% CI)	Absolute (95% CI)		
**Difference in changes of FBG post intervention value within 4 months between CL supplementation and control group**												
23	randomised trials	not serious	serious[a]	not serious	not serious	none	896	931	-	MD –**8.129 lower** (–12.975 lower to –4.084 lower)	⨁⨁⨁◯ Moderate	CRITICAL
**Difference in changes of FBG change from baseline within 4 months between CL supplementation and control group**												
14	randomised trials	not serious	serious[a]	not serious	not serious	none	482	557	-	MD –**8.833 lower** (–13.907 lower to –3.758 lower)	⨁⨁⨁◯ Moderate	CRITICAL
**Difference in changes of HbA1C post intervention value within 4 months between CL supplementation and control group**												
21	randomised trials	not serious	serious[a]	not serious	not serious	none	813	848	-	MD –**0.134 lower** (–0.304 lower to –0.037 lower)	⨁⨁⨁◯ Moderate	CRITICAL
**Difference in changes of A1C change from baseline within 4 months between CL supplementation and control group**												
11	randomised trials	not serious	serious[a]	not serious	not serious	none	389	484	-	MD –**0.517 lower** (–0.707 lower to –0.327 lower)	⨁⨁⨁◯ Moderate	CRITICAL

a. large I-square 75.8%

Abbreviations: CI, confidence interval; CL, *Curcuma longa*; MD, mean difference.
