# Supplementary material for: Effects of turmeric (Curcuma longa) supplementation on glucose metabolism in diabetes mellitus and metabolic syndrome: An umbrella review and updated meta-analysis
Source: PLoS One. 2023 Jul 20;18(7):e0288997. doi: 10.1371/journal.pone.0288997 (PMC10359013; doi:10.1371/journal.pone.0288997)
Supplement: S1 File — (ZIP) [file pone.0288997.s002.zip › Table S2.pdf]

Table S2. General characteristics of treatment options.

Code	Forms of *C. longa* preparations	*C. longa* products
1	Whole	Turmeric alone
		Turmeric capsule
		Turmeric powder
		Unformulated curcumin capsules (UC)
		Turmeric supplementation
2	Extract	BCM95/Curcugreen capsule
		C. longa extract
		Curcumin capsule
		Curcumin extract capsule
		Curcumin supplement
		Curcuminoids capsule
		NCB-02
3	Bioav-enhanced	C3 Complex® + Bioperine®
		Capsules of long turmeric and piperine (CURPI group)
		Meriva® curcumin tablet (CC)
		Nano curcumin capsules
		Phospholipidated curcumin (PC) capsules (absorption-enhanced curcumin capsules)
		SinaCurcumin® (soft gel of Nano-curcumin)
		Nanocurcumin tablet
		Soft gel of nano-micelle curcumin
		Theracurmin®, a highly absorbable curcumin preparation (fine granules of curcumin with a coating)
1D	Whole	Turmeric powder + metformin therapy
1int	Whole	Turmeric supplementation + aerobic training
2int	Extract	Curcumin and Zinc
3int	Bioav-enhanced	Meriva® + fish oil (CC-FO)
