# Supplementary material for: Effects of turmeric (Curcuma longa) supplementation on glucose metabolism in diabetes mellitus and metabolic syndrome: An umbrella review and updated meta-analysis
Source: PLoS One. 2023 Jul 20;18(7):e0288997. doi: 10.1371/journal.pone.0288997 (PMC10359013; doi:10.1371/journal.pone.0288997)
Supplement: S1 File — (ZIP) [file pone.0288997.s002.zip › Table S4.pdf]

Table S4. Quality assessment of included studies in SRMAs

Study name	AMSTAR-2 rating
Altobelli E, 2021[1]	Critically Low
Ashtary-Larky D, 2021[2]	Critically Low
Azhdari M, 2019[3]	Critically Low
de Melo ISV, 2018[4]	Critically Low
Gorabi 2022[5]	Critically Low
Huang J, 2019[6]	Critically Low
Macena ML, 2022[7]	Low
Panahi Y, 2015[8]	Critically Low
Tabrizi R, 2018[9]	Critically Low
Tian J, 2022[10]	Critically Low
Yuan F, 2019[11]	Critically Low
Yuan F, 2022[12]	Critically Low
Zhang T, 2021[13]	Critically Low
Zheng ZH, 2021[14]	Critically Low

S2. References

1. Altobelli E, Angeletti PM, Marziliano C, Mastrodomenico M, Giuliani AR, Petrocelli R. Potential therapeutic effects of curcumin on glycemic and lipid profile in uncomplicated type 2 diabetes—a meta-analysis of randomized controlled trial. Nutrients. 2021;13(2):1-13.
2. Ashtary-Larky D, Rezaei Kelishadi M, Bagheri R, Moosavian SP, Wong A, Davoodi SH, et al. The effects of nano-curcumin supplementation on risk factors for cardiovascular disease: a GRADE-assessed systematic review and meta-analysis of clinical trials. Antioxidants. 2021;10(7):1015.
3. Azhdari M, Karandish M, Mansoori A. Metabolic benefits of curcumin supplementation in patients with metabolic syndrome: a systematic review and meta-analysis of randomized controlled trials. Phytotherapy Research. 2019;33(5):1289-301.
4. de Melo ISV, Dos Santos AF, Bueno NB. Curcumin or combined curcuminoids are effective in lowering the fasting blood glucose concentrations of individuals with dysglycemia: systematic review and meta-analysis of randomized controlled trials. Pharmacological Research. 2018;128:137-44.
5. Gorabi AM, Abbasifard M, Imani D, Aslani S, Razi B, Alizadeh S, et al. Effect of curcumin on C-reactive protein as a biomarker of systemic inflammation: an updated meta-analysis of randomized controlled trials. Phytotherapy Research. 2022;36(1):85-97.
6. Huang J, Qin S, Huang L, Tang Y, Ren H, Hu H. Efficacy and safety of Rhizoma *Curcumea longae* with respect to improving the glucose metabolism of patients at risk for cardiovascular disease: a meta-analysis of randomised controlled trials. Journal of Human Nutrition and Dietetics 2019;32(5):591-606.
7. Macena ML, Nunes LFDS, da Silva AF, Pureza IROM, Praxedes DRS, Santos JCF, et al. Effects of dietary polyphenols in the glycemic, renal, inflammatory, and oxidative stress biomarkers in diabetic nephropathy: a systematic review with meta-analysis of randomized controlled trials. Nutrition Reviews. 2022.
8. Panahi Y, Hosseini MS, Khalili N, Naimi E, Majeed M, Sahebkar A. Antioxidant and anti-inflammatory effects of curcuminoid-piperine combination in subjects with metabolic syndrome: a randomized controlled trial and an updated meta-analysis. Clinical Nutrition. 2015;34(6):1101-8.
9. Tabrizi R, Vakili S, Lankarani KB, Akbari M, Mirhosseini N, Ghayour-Mobarhan M, et al. The effects of curcumin on glycemic control and lipid profiles among patients with metabolic syndrome and related disorders: a systematic review and metaanalysis of randomized controlled trials. Current Pharmaceutical Design. 2018;24(27):3184-99.
10. Tian J, Feng B, Tian Z. The effect of curcumin on lipid profile and glycemic status of patients with type 2 diabetes mellitus: a systematic review and meta-analysis. Evidence-Based Complementary and Alternative Medicine. 2022;2022:8278744.
11. Yuan F, Dong H, Gong J, Wang D, Hu M, Huang W, et al. A systematic review and meta-analysis of randomized controlled trials on the effects of turmeric and curcuminoids on blood lipids in adults with metabolic diseases. Advances in Nutrition. 2019;10(5):791-802.
12. Yuan F, Wu W, Ma L, Wang D, Hu M, Gong J, et al. Turmeric and curcuminiods ameliorate disorders of glycometabolism among subjects with metabolic diseases: a systematic review and meta-analysis of randomized controlled trials. Pharmacological Research. 2022;177:106121.
13. Zhang T, He Q, Liu Y, Chen Z, Hu H. Efficacy and safety of curcumin supplement on improvement of insulin resistance in people with type 2 diabetes mellitus: a systematic review and meta-analysis of randomized controlled trials. Evidence-Based Complementary and Alternative Medicine. 2021;2021.
14. Zheng ZH, Pan HJ, Zhao ZN, Yuan H, Li WY, Zhang BG, et al. Meta-analysis of efficacy of curcumin in the treatment of complications of type II diabetes mellitus. Chinese Pharmaceutical Journal. 2021;56(6):489-96.
15. Adab Z, Eghtesadi S, Vafa M-R, Heydari I, Shojaii A, Haqqani H, et al. Effect of turmeric on glycemic status, lipid profile, hs-CRP, and total antioxidant capacity in hyperlipidemic type 2 diabetes mellitus patients. Phytotherapy Research. 2019;33(4):1173-81.
16. Adibian M, Hodaei H, Nikpayam O, Sohrab G, Hekmatdoost A, Hedayati M. The effects of curcumin supplementation on high-sensitivity C-reactive protein, serum adiponectin, and lipid profile in patients with type 2 diabetes: a randomized, double-blind, placebo-controlled trial. Phytotherapy Research. 2019;33(5):1374-83.
17. Alidadi M, Sahebkar A, Eslami S, Vakilian F, Jarahi L, Alinezhad-Namaghi M, et al. The effect of curcumin supplementation on pulse wave velocity in patients with metabolic syndrome: a randomized, double-blind, placebo-controlled trial. Pharmacological properties of plant-derived natural products and implications for human health. 1308: Springer, Cham.; 2021. p. 1-11.
18. Amin F, Islam N, Anila N, Gilani AH. Clinical efficacy of the co-administration of turmeric and black seeds (Kalongi) in metabolic syndrome–a double blind randomized controlled trial–TAK-MetS trial. Complementary Therapies in Medicine. 2015;23(2):165-74.
19. Asadi S, Gholami MS, Siassi F, Qorbani M, Khamoshian K, Sotoudeh G. Nano curcumin supplementation reduced the severity of diabetic sensorimotor polyneuropathy in patients with type 2 diabetes

mellitus: a randomized double-blind placebo-controlled clinical trial. Complementary Therapies in Medicine. 2019;43:253-60.
20. Bateni Z, Rahimi HR, Hedayati M, Afsharian S, Goudarzi R, Sohrab G. The effects of nano-curcumin supplementation on glycemic control, blood pressure, lipid profile, and insulin resistance in patients with the metabolic syndrome: a randomized, double-blind clinical trial. Phytotherapy Research. 2021;35(7):3945-53.
21. Bateni Z, Behrouz V, Rahimi HR, Hedayati M, Afsharian S, Sohrab G. Effects of nano-curcumin supplementation on oxidative stress, systemic inflammation, adiponectin, and NF-κB in patients with metabolic syndrome: a randomized, double-blind clinical trial. Journal of Herbal Medicine. 2022;31.
22. Chuengsamarn S, Rattanamongkolgul S, Luechapudiporn R, Phisalaphong C, Jirawatnotai S. Curcumin extract for prevention of type 2 diabetes. Diabetes Care. 2012;35(11):2121-7.
23. Chuengsamarn S, Rattanamongkolgul S, Phonrat B, Tungtrongchitr R, Jirawatnotai S. Reduction of atherogenic risk in patients with type 2 diabetes by curcuminoid extract: a randomized controlled trial. Journal of Nutritional Biochemistry. 2014;25(2):144-50.
24. Darmian MA, Hoseini R, Amiri E, Golshani S. How combined and separate aerobic training and turmeric supplementation alter lipid profile and glycemic status? A clinical trial in middle-aged females with type 2 diabetes and hyperlipidemia. International Cardiovascular Research Journal. 2021;15(3):111-8.
25. Darmian MA, Hoseini R, Amiri E, Golshani S. Downregulated hs-CRP and MAD, upregulated GSH and TAC, and improved metabolic status following combined exercise and turmeric supplementation: a clinical trial in middle-aged women with hyperlipidemic type 2 diabetes. Journal of Diabetes & Metabolic Disorders. 2022;21(1):275-83.
26. Dastani M, Rahimi HR, Askari VR, Jaafari MR, Jarahi L, Yadollahi A, et al. Three months of combination therapy with nano-curcumin reduces the inflammation and lipoprotein (a) in type 2 diabetic patients with mild to moderate coronary artery disease: Evidence of a randomized, double-blinded, placebo-controlled clinical trial. Biofactors. 2022.
27. Funamoto M, Shimizu K, Sunagawa Y, Katanasaka Y, Miyazaki Y, Kakeya H, et al. Effects of highly absorbable curcumin in patients with impaired glucose tolerance and non-insulin-dependent diabetes mellitus. Journal of Diabetes Research. 2019;2019.
28. Hodaei H, Adibian M, Nikpayam O, Hedayati M, Sohrab G. The effect of curcumin supplementation on anthropometric indices, insulin resistance and oxidative stress in patients with type 2 diabetes: a randomized, double-blind clinical trial. Diabetology and Metabolic Syndrome. 2019;11(1):1-8.
29. Jiménez-Osorio AS, García-Niño WR, González-Reyes S, Álvarez-Mejía AE, Guerra-León S, Salazar-Segovia J, et al. The effect of dietary supplementation with curcumin on redox status and Nrf2 activation in patients with nondiabetic or diabetic proteinuric chronic kidney disease: a pilot study. Journal of Renal Nutrition. 2016;26(4):237-44.
30. Karandish M, Mozaffari-Khosravi H, Mohammadi SM, Cheraghian B, Azhdari M. The effect of curcumin and zinc co-supplementation on glycemic parameters in overweight or obese prediabetic subjects: a phase 2 randomized, placebo-controlled trial with a multi-arm, parallel-group design. Phytotherapy Research. 2021;35(8):4377-87.
31. Karandish M, Mozaffari-Khosravi H, Mohammadi SM, Cheraghian B, Azhdari M. Curcumin and zinc co-supplementation along with a loss-weight diet can improve lipid profiles in subjects with prediabetes: a multi-arm, parallel-group, randomized, double-blind placebo-controlled phase 2 clinical trial. Diabetology & Metabolic Syndrome. 2022;14(1):22.
32. Khajehdehi P, Pakfetrat M, Javidnia K, Azad F, Malekmakan L, Nasab MH, et al. Oral supplementation of turmeric attenuates proteinuria, transforming growth factor-β and interleukin-8 levels in patients with overt type 2 diabetic nephropathy: a randomized, double-blind and placebo-controlled study. Scandinavian Journal of Urology and Nephrology. 2011;45(5):365-70.
33. Maithili Karpaga Selvi N, Sridhar MG, Swaminathan RP, Sripradha R. Efficacy of turmeric as adjuvant therapy in type 2 diabetic patients. Indian Journal of Clinical Biochemistry. 2015;30(2):180-6.
34. Mokhtari M, Razzaghi R, Momen-Heravi M. The effects of curcumin intake on wound healing and metabolic status in patients with diabetic foot ulcer: a randomized, double-blind, placebo-controlled trial. Phytotherapy Research. 2021;35(4):2099-107.
35. Na LX, Li Y, Pan HZ, Zhou XL, Sun DJ, Meng M, et al. Curcuminoids exert glucose-lowering effect in type 2 diabetes by decreasing serum free fatty acids: a double-blind, placebo-controlled trial. Molecular Nutrition and Food Research. 2013;57(9):1569-77.
36. Neta JFF, Veras VS, Sousa DF, Cunha MDCDSO, Queiroz MVO, Neto JCGL, et al. Effectiveness of the piperine-supplemented *Curcuma longa* L. in metabolic control of patients with type 2 diabetes: a randomised double-blind placebo-controlled clinical trial. International Journal of Food Sciences and Nutrition. 2021;72(7):968-77.

37. Panahi Y, Khalili N, Hosseini MS, Abbasinazari M, Sahebkar A. Lipid-modifying effects of adjunctive therapy with curcuminoids-piperine combination in patients with metabolic syndrome: results of a randomized controlled trial. Complementary Therapies in Medicine. 2014;22(5):851-7.
38. Panahi Y, Khalili N, Sahebi E, Namazi S, Reiner, Zcaron, et al. Curcuminoids modify lipid profile in type 2 diabetes mellitus: a randomized controlled trial. Complementary Therapies in Medicine. 2017;33:1-5.
39. Panahi Y, Khalili N, Sahebi E, Namazi S, Simental-Mendía LE, Majeed M, et al. Effects of curcuminoids plus piperine on glycemic, hepatic and inflammatory biomarkers in patients with type 2 diabetes mellitus: a randomized double-blind placebo-controlled trial. Drug Research. 2018;68(7):403-9.
40. Rahimi HR, Mohammadpour AH, Dastani M, Jaafari MR, Abnous K, Mobarhan MG, et al. The effect of nano-curcumin on HbA1c, fasting blood glucose, and lipid profile in diabetic subjects: a randomized clinical trial. Avicenna Journal of Phytomedicine. 2016;6(5):567-77.
41. Saberi-Karimian M, Parizadeh SMR, Ghayour-Mobarhan M, Salahshooh MM, Dizaji BF, Safarian H, et al. Evaluation of the effects of curcumin in patients with metabolic syndrome. Comparative Clinical Pathology. 2018;27(3):555-63.
42. Sousa DF, Araújo M, de Mello VD, Damasceno M, Freitas R. Cost-effectiveness of passion fruit albedo versus turmeric in the glycemic and lipaemic control of people with type 2 diabetes: randomized clinical trial. Journal of the American College of Nutrition. 2021;40(8):679-88.
43. Thota RN, Acharya SH, Garg ML. Curcumin and/or omega-3 polyunsaturated fatty acids supplementation reduces insulin resistance and blood lipids in individuals with high risk of type 2 diabetes: a randomised controlled trial. Lipids in Health and Disease. 2019;18(1):1-11.
44. Usharani P, Mateen AA, Naidu MU, Raju YS, Chandra N. Effect of NCB-02, atorvastatin and placebo on endothelial function, oxidative stress and inflammatory markers in patients with type 2 diabetes mellitus: a randomized, parallel-group, placebo-controlled, 8-week study. Drugs in R&D. 2008;9(4):243-50.
45. Vanaie A, Shahidi S, Iraj B, Siadat ZD, Kabirzade M, Shakiba F, et al. Curcumin as a major active component of turmeric attenuates proteinuria in patients with overt diabetic nephropathy. Journal of Research in Medical Sciences. 2019;24:77.
46. Yang YS, Su YF, Yang HW, Lee YH, Chou JI, Ueng KC. Lipid-lowering effects of curcumin in patients with metabolic syndrome: a randomized, double-blind, placebo-controlled trial. Phytotherapy Research. 2014;28(12):1770-7.
