# Supplementary material for: Effects of turmeric (Curcuma longa) supplementation on glucose metabolism in diabetes mellitus and metabolic syndrome: An umbrella review and updated meta-analysis
Source: PLoS One. 2023 Jul 20;18(7):e0288997. doi: 10.1371/journal.pone.0288997 (PMC10359013; doi:10.1371/journal.pone.0288997)
Supplement: S1 File — (ZIP) [file pone.0288997.s002.zip › Table S6.pdf]

Table S6. Description of characteristic of the included individual studies.

Study	Forms of CL preparation	CL preparation dose (mg/day)	Amount of APIs (mg/day)		Male (%)	Duration of outcome measurement (wks)	DM	pre-DM	Metabolic Syndrome	Another underlying disease	Exercise	Baseline standard treatment/medication/another supplement
			curcuminoids	curcumin								
Adab Z, 2019[15]	Whole	2100	NI	NI	48	8	Yes	No	No	Yes	No	Blood sugar-lowering agents (Metformin, Glibenclamide, Metformin, Gliclazide) Blood lipid-lowering agents (Atorvastatin, Cholestyramine, Fenofibrate or Gemfibrozil)
Adibian M, 2019[16]	Extract	1500	1320	1041	50	10	Yes	No	No	No	No	Antihyperglycemic drugs (metformin, gelibenclamide, Metformin + Gelibenclamide, None) Anti-hypertensive drugs, Anti-hyperlipidemic drugs
Alidadi, M, 2021[17]	Extract	500	475	NI	50	12	No	No	Yes	No	No	None
Amin F, 2015[18]	Whole	2400	NI	NI	100	8	No	Yes	Yes	Yes	No	NI
Asadi S, 2019[19]	Bioav-enhanced	80	NI	57.6	12.5	8	Yes	No	No	No	No	Metformin, Metformin+Glibenglamid, Atorvastatin+Metformin, Atorvastatin+Metformin+Glibenglamid
Bateni Z, 2021-2022[20,21]	Bioav-enhanced	80	NI	80	23.3	12	No	No	Yes	No	No	Antihypertensive drugs: ACE inhibitors, Angiotensin-2, Receptor blockers, Beta blockers, other drugs<br><br>Antihyperlipidemic drugs: Statins, Fibrates<br><br>Hypoglycemic agents: Biguanides, Sulfonylureas, other drugs
Chuengsamarn S, 2012[22]	Extract	NI	1500	NI	35.1	12	No	Yes	No	Yes	No	No baseline treatment (excluded all of patients who were taking any other medicines)
Chuengsamarn, S, 2014[23]	Extract	NI	1500	NI	45.5	12	Yes	No	No	Yes	No	No baseline treatment (excluded all of patients who were taking any other medicines)
Darmian MA, 2021–2022[24,25]	Whole	2100	NI	NI	0	8	Yes	No	Yes	Yes	Yes	Metformin, Glibenclamide, Metformin + Glibenclamide, Gliclazide, Atorvastatin, Cholestyramine, Fenofibrate or Gemfibrozil
Dastani M, 2022[26]	Bioav-enhanced	80	NI	80	39.1	12	No	No	Yes	No	Yes	anti-diabetic drugs, angiotensin- converting enzyme inhibitors, selective beta-blockers, anti- platelet medications (aspirin and clopidogrel), and high doses of statin (atorvastatin 80 mg)

Study	Forms of CL preparation	CL preparation dose (mg/day)	Amount of APIs (mg/day)		Male (%)	Duration of outcome measurement (wks)	DM	pre-DM	Metabolic Syndrome	Another underlying disease	Exercise	Baseline standard treatment/medication/another supplement
			curcuminoids	curcumin								
Funamoto M, 2019[27]	Bioav-enhanced	180	NI	18	68.8	12	Yes	Yes	No	No	No	Antihypertensive drug, α-Glucosidase inhibitor, DPP-4inhibitor, Sulfonylurea<br>Biguanide, Glinide, Thiazolidinediones
Hodaei H, 2019[28]	Extract	1500	1320	1041	50.9	10	Yes	No	No	No	No	1. Anti-hyperglycemic drugs: metformin, Gelibenclamide, Gelibenclamide + metformin<br>2. Anti-hyperlipidemic drugs<br>3. Anti-hypertensive drugs<br>4. None
Jiménez-Osorio AS, 2016[29]	Whole	NI	NI	320	60.4	8	Yes	No	No	Yes	No	NI
Karandish M, 2021-2022[30,31]	Extract	500	>475	>325	31.7	12	No	Yes	No	Yes	No	NI
Khajehdehi P, 2011[32]	Whole	1500	NI	66.3	55	8	Yes	No	No	Yes	No	ACE-I and/or angiotensin receptor blockers
Maithili KSN, 2015[33]	Whole	2000	NI	46	100	4	Yes	No	No	No	No	NI
Mokhtari M, 2021[34]	Bioav-enhanced	80	NI	80	78	12	Yes	No	No	Yes	No	All subjects underwent a similar treatment protocol based on the international consensus on the diabetic foot, which included ciprofloxacin (Cipro) 400 mg IV twice a day plus clindamycin 900 mg IV three times per day for 3 weeks.
Na LX, 2013[35]	Extract	300	292.47	108.18	49	12	Yes	No	No	Yes	No	1. Oral hypoglycemic agents, insulin or both 2. Lipid-lowering drugs 3. antihypertensive medications (Angiotensin converting enzyme inhibitor, Angiotensin II receptor blocker, or both Other antihypertensive agents)
Neta JFF, 2021[36]	Extract	500	450	NI	25.5	16	Yes	No	No	No	No	Sulfonylureas and biguanides
Panahi Y, 2014–2015[37]	Bioav-enhanced	1000	>950	NI	50	8	No	No	Yes	No	No	All subjects received standard of care for MS including lifestyle (physical activity, weight loss, and avoiding smoking and alcohol) and dietary (adherence to a heart-healthy diet) modifications, and pharmacological interventions. Pharmacotherapy for MS included the use of lipid-lowering agents (statins, fibrates, and low-dose niacin), antihypertensive agents

Study	Forms of CL preparation	CL preparation dose (mg/day)	Amount of APIs (mg/day)		Male (%)	Duration of outcome measurement (wks)	DM	pre-DM	Metabolic Syndrome	Another underlying disease	Exercise	Baseline standard treatment/medication/another supplement
			curcuminoids	curcumin								
												(angiotensin converting enzyme inhibitors and angiotensin receptor blockers) and oral hypoglycemic agents (metformin and glybenclamide)
Panahi Y, 2017–2018[38,39]	Bioav-enhanced	1000	>950	NI	51	12	Yes	No	No	No	No	NI
Rahimi HR, 2016[40]	Bioav-enhanced	80	NI	80	44.3	12	Yes	No	No	Yes	No	All other necessary medications were given to subjects
Saberi-Karimian M, 2018[41]	Bioav-enhanced	1000	NI	200	NI	6	No	No	Yes	No	No	NI
	Whole	1000	NI	NI								
Sousa DFD, 2021[42]	Bioav-enhanced	500	NI	NI	21.35	16	Yes	No	No	No	No	
Thota RN, 2019[43]	Bioav-enhanced	1000	>200	180	40.6	12	No	Yes	No	No	No	
Usharani P, 2008[44]	Extract	600	NI	300	NI	8	Yes	No	No	No	No	NI
Vanaie A, 2022[45]	Extract	1500	1500	NI	58.7	16	Yes	No	No	Yes	No	Oral antidiabetic drugs or insulin, ACEi, ARBs
Yang Y, 2014[46]	Extract	1950	1795.5	NI	44.6	12	No	No	Yes	No	No	NI

Abbreviations: APIs, active pharmaceutical ingredients; Bioav-enhanced, bioavailability-enhanced curcumin preparation; CL, *Curcuma longa*; DM, diabetes; Extract, curcumin-extract preparation; MetS, metabolic syndromes; NI, not indicated; pre-DM, prediabetes; Whole, whole-phytochemicals curcumin preparation, wks, weeks.

S2. References

1. Altobelli E, Angeletti PM, Marziliano C, Mastrodomenico M, Giuliani AR, Petrocelli R. Potential therapeutic effects of curcumin on glycemic and lipid profile in uncomplicated type 2 diabetes—a meta-analysis of randomized controlled trial. Nutrients. 2021;13(2):1-13.
2. Ashtary-Larky D, Rezaei Kelishadi M, Bagheri R, Moosavian SP, Wong A, Davoodi SH, et al. The effects of nano-curcumin supplementation on risk factors for cardiovascular disease: a GRADE-assessed systematic review and meta-analysis of clinical trials. Antioxidants. 2021;10(7):1015.
3. Azhdari M, Karandish M, Mansoori A. Metabolic benefits of curcumin supplementation in patients with metabolic syndrome: a systematic review and meta-analysis of randomized controlled trials. Phytotherapy Research. 2019;33(5):1289-301.
4. de Melo ISV, Dos Santos AF, Bueno NB. Curcumin or combined curcuminoids are effective in lowering the fasting blood glucose concentrations of individuals with dysglycemia: systematic review and meta-analysis of randomized controlled trials. Pharmacological Research. 2018;128:137-44.
5. Gorabi AM, Abbasifard M, Imani D, Aslani S, Razi B, Alizadeh S, et al. Effect of curcumin on C-reactive protein as a biomarker of systemic inflammation: an updated meta-analysis of randomized controlled trials. Phytotherapy Research. 2022;36(1):85-97.
6. Huang J, Qin S, Huang L, Tang Y, Ren H, Hu H. Efficacy and safety of Rhizoma *Curcumea longae* with respect to improving the glucose metabolism of patients at risk for cardiovascular disease: a meta-analysis of randomised controlled trials. Journal of Human Nutrition and Dietetics 2019;32(5):591-606.
7. Macena ML, Nunes LFDS, da Silva AF, Pureza IROM, Praxedes DRS, Santos JCF, et al. Effects of dietary polyphenols in the glycemic, renal, inflammatory, and oxidative stress biomarkers in diabetic nephropathy: a systematic review with meta-analysis of randomized controlled trials. Nutrition Reviews. 2022.
8. Panahi Y, Hosseini MS, Khalili N, Naimi E, Majeed M, Sahebkar A. Antioxidant and anti-inflammatory effects of curcuminoid-piperine combination in subjects with metabolic syndrome: a randomized controlled trial and an updated meta-analysis. Clinical Nutrition. 2015;34(6):1101-8.
9. Tabrizi R, Vakili S, Lankarani KB, Akbari M, Mirhosseini N, Ghayour-Mobarhan M, et al. The effects of curcumin on glycemic control and lipid profiles among patients with metabolic syndrome and related disorders: a systematic review and metaanalysis of randomized controlled trials. Current Pharmaceutical Design. 2018;24(27):3184-99.
10. Tian J, Feng B, Tian Z. The effect of curcumin on lipid profile and glycemic status of patients with type 2 diabetes mellitus: a systematic review and meta-analysis. Evidence-Based Complementary and Alternative Medicine. 2022;2022:8278744.
11. Yuan F, Dong H, Gong J, Wang D, Hu M, Huang W, et al. A systematic review and meta-analysis of randomized controlled trials on the effects of turmeric and curcuminoids on blood lipids in adults with metabolic diseases. Advances in Nutrition. 2019;10(5):791-802.
12. Yuan F, Wu W, Ma L, Wang D, Hu M, Gong J, et al. Turmeric and curcuminiods ameliorate disorders of glycometabolism among subjects with metabolic diseases: a systematic review and meta-analysis of randomized controlled trials. Pharmacological Research. 2022;177:106121.
13. Zhang T, He Q, Liu Y, Chen Z, Hu H. Efficacy and safety of curcumin supplement on improvement of insulin resistance in people with type 2 diabetes mellitus: a systematic review and meta-analysis of randomized controlled trials. Evidence-Based Complementary and Alternative Medicine. 2021;2021.
14. Zheng ZH, Pan HJ, Zhao ZN, Yuan H, Li WY, Zhang BG, et al. Meta-analysis of efficacy of curcumin in the treatment of complications of type II diabetes mellitus. Chinese Pharmaceutical Journal. 2021;56(6):489-96.
15. Adab Z, Eghtesadi S, Vafa M-R, Heydari I, Shojaii A, Haqqani H, et al. Effect of turmeric on glycemic status, lipid profile, hs-CRP, and total antioxidant capacity in hyperlipidemic type 2 diabetes mellitus patients. Phytotherapy Research. 2019;33(4):1173-81.
16. Adibian M, Hodaei H, Nikpayam O, Sohrab G, Hekmatdoost A, Hedayati M. The effects of curcumin supplementation on high-sensitivity C-reactive protein, serum adiponectin, and lipid profile in patients with type 2 diabetes: a randomized, double-blind, placebo-controlled trial. Phytotherapy Research. 2019;33(5):1374-83.
17. Alidadi M, Sahebkar A, Eslami S, Vakilian F, Jarahi L, Alinezhad-Namaghi M, et al. The effect of curcumin supplementation on pulse wave velocity in patients with metabolic syndrome: a randomized, double-blind, placebo-controlled trial. Pharmacological properties of plant-derived natural products and implications for human health. 1308: Springer, Cham.; 2021. p. 1-11.
18. Amin F, Islam N, Anila N, Gilani AH. Clinical efficacy of the co-administration of turmeric and black seeds (Kalongi) in metabolic syndrome–a double blind randomized controlled trial–TAK-MetS trial. Complementary Therapies in Medicine. 2015;23(2):165-74.
19. Asadi S, Gholami MS, Siassi F, Qorbani M, Khamoshian K, Sotoudeh G. Nano curcumin supplementation reduced the severity of diabetic sensorimotor polyneuropathy in patients with type 2 diabetes

mellitus: a randomized double-blind placebo-controlled clinical trial. Complementary Therapies in Medicine. 2019;43:253-60.
20. Bateni Z, Rahimi HR, Hedayati M, Afsharian S, Goudarzi R, Sohrab G. The effects of nano-curcumin supplementation on glycemic control, blood pressure, lipid profile, and insulin resistance in patients with the metabolic syndrome: a randomized, double-blind clinical trial. Phytotherapy Research. 2021;35(7):3945-53.
21. Bateni Z, Behrouz V, Rahimi HR, Hedayati M, Afsharian S, Sohrab G. Effects of nano-curcumin supplementation on oxidative stress, systemic inflammation, adiponectin, and NF-κB in patients with metabolic syndrome: a randomized, double-blind clinical trial. Journal of Herbal Medicine. 2022;31.
22. Chuengsamarn S, Rattanamongkolgul S, Luechapudiporn R, Phisalaphong C, Jirawatnotai S. Curcumin extract for prevention of type 2 diabetes. Diabetes Care. 2012;35(11):2121-7.
23. Chuengsamarn S, Rattanamongkolgul S, Phonrat B, Tungtrongchitr R, Jirawatnotai S. Reduction of atherogenic risk in patients with type 2 diabetes by curcuminoid extract: a randomized controlled trial. Journal of Nutritional Biochemistry. 2014;25(2):144-50.
24. Darmian MA, Hoseini R, Amiri E, Golshani S. How combined and separate aerobic training and turmeric supplementation alter lipid profile and glycemic status? A clinical trial in middle-aged females with type 2 diabetes and hyperlipidemia. International Cardiovascular Research Journal. 2021;15(3):111-8.
25. Darmian MA, Hoseini R, Amiri E, Golshani S. Downregulated hs-CRP and MAD, upregulated GSH and TAC, and improved metabolic status following combined exercise and turmeric supplementation: a clinical trial in middle-aged women with hyperlipidemic type 2 diabetes. Journal of Diabetes & Metabolic Disorders. 2022;21(1):275-83.
26. Dastani M, Rahimi HR, Askari VR, Jaafari MR, Jarahi L, Yadollahi A, et al. Three months of combination therapy with nano-curcumin reduces the inflammation and lipoprotein (a) in type 2 diabetic patients with mild to moderate coronary artery disease: Evidence of a randomized, double-blinded, placebo-controlled clinical trial. Biofactors. 2022.
27. Funamoto M, Shimizu K, Sunagawa Y, Katanasaka Y, Miyazaki Y, Kakeya H, et al. Effects of highly absorbable curcumin in patients with impaired glucose tolerance and non-insulin-dependent diabetes mellitus. Journal of Diabetes Research. 2019;2019.
28. Hodaei H, Adibian M, Nikpayam O, Hedayati M, Sohrab G. The effect of curcumin supplementation on anthropometric indices, insulin resistance and oxidative stress in patients with type 2 diabetes: a randomized, double-blind clinical trial. Diabetology and Metabolic Syndrome. 2019;11(1):1-8.
29. Jiménez-Osorio AS, García-Niño WR, González-Reyes S, Álvarez-Mejía AE, Guerra-León S, Salazar-Segovia J, et al. The effect of dietary supplementation with curcumin on redox status and Nrf2 activation in patients with nondiabetic or diabetic proteinuric chronic kidney disease: a pilot study. Journal of Renal Nutrition. 2016;26(4):237-44.
30. Karandish M, Mozaffari-Khosravi H, Mohammadi SM, Cheraghian B, Azhdari M. The effect of curcumin and zinc co-supplementation on glycemic parameters in overweight or obese prediabetic subjects: a phase 2 randomized, placebo-controlled trial with a multi-arm, parallel-group design. Phytotherapy Research. 2021;35(8):4377-87.
31. Karandish M, Mozaffari-Khosravi H, Mohammadi SM, Cheraghian B, Azhdari M. Curcumin and zinc co-supplementation along with a loss-weight diet can improve lipid profiles in subjects with prediabetes: a multi-arm, parallel-group, randomized, double-blind placebo-controlled phase 2 clinical trial. Diabetology & Metabolic Syndrome. 2022;14(1):22.
32. Khajehdehi P, Pakfetrat M, Javidnia K, Azad F, Malekmakan L, Nasab MH, et al. Oral supplementation of turmeric attenuates proteinuria, transforming growth factor-β and interleukin-8 levels in patients with overt type 2 diabetic nephropathy: a randomized, double-blind and placebo-controlled study. Scandinavian Journal of Urology and Nephrology. 2011;45(5):365-70.
33. Maithili Karpaga Selvi N, Sridhar MG, Swaminathan RP, Sripradha R. Efficacy of turmeric as adjuvant therapy in type 2 diabetic patients. Indian Journal of Clinical Biochemistry. 2015;30(2):180-6.
34. Mokhtari M, Razzaghi R, Momen-Heravi M. The effects of curcumin intake on wound healing and metabolic status in patients with diabetic foot ulcer: a randomized, double-blind, placebo-controlled trial. Phytotherapy Research. 2021;35(4):2099-107.
35. Na LX, Li Y, Pan HZ, Zhou XL, Sun DJ, Meng M, et al. Curcuminoids exert glucose-lowering effect in type 2 diabetes by decreasing serum free fatty acids: a double-blind, placebo-controlled trial. Molecular Nutrition and Food Research. 2013;57(9):1569-77.
36. Neta JFF, Veras VS, Sousa DF, Cunha MDCDSO, Queiroz MVO, Neto JCGL, et al. Effectiveness of the piperine-supplemented *Curcuma longa* L. in metabolic control of patients with type 2 diabetes: a randomised double-blind placebo-controlled clinical trial. International Journal of Food Sciences and Nutrition. 2021;72(7):968-77.

37. Panahi Y, Khalili N, Hosseini MS, Abbasinazari M, Sahebkar A. Lipid-modifying effects of adjunctive therapy with curcuminoids-piperine combination in patients with metabolic syndrome: results of a randomized controlled trial. Complementary Therapies in Medicine. 2014;22(5):851-7.
38. Panahi Y, Khalili N, Sahebi E, Namazi S, Reiner, Zcaron, et al. Curcuminoids modify lipid profile in type 2 diabetes mellitus: a randomized controlled trial. Complementary Therapies in Medicine. 2017;33:1-5.
39. Panahi Y, Khalili N, Sahebi E, Namazi S, Simental-Mendía LE, Majeed M, et al. Effects of curcuminoids plus piperine on glycemic, hepatic and inflammatory biomarkers in patients with type 2 diabetes mellitus: a randomized double-blind placebo-controlled trial. Drug Research. 2018;68(7):403-9.
40. Rahimi HR, Mohammadpour AH, Dastani M, Jaafari MR, Abnous K, Mobarhan MG, et al. The effect of nano-curcumin on HbA1c, fasting blood glucose, and lipid profile in diabetic subjects: a randomized clinical trial. Avicenna Journal of Phytomedicine. 2016;6(5):567-77.
41. Saberi-Karimian M, Parizadeh SMR, Ghayour-Mobarhan M, Salahshooh MM, Dizaji BF, Safarian H, et al. Evaluation of the effects of curcumin in patients with metabolic syndrome. Comparative Clinical Pathology. 2018;27(3):555-63.
42. Sousa DF, Araújo M, de Mello VD, Damasceno M, Freitas R. Cost-effectiveness of passion fruit albedo versus turmeric in the glycemic and lipaemic control of people with type 2 diabetes: randomized clinical trial. Journal of the American College of Nutrition. 2021;40(8):679-88.
43. Thota RN, Acharya SH, Garg ML. Curcumin and/or omega-3 polyunsaturated fatty acids supplementation reduces insulin resistance and blood lipids in individuals with high risk of type 2 diabetes: a randomised controlled trial. Lipids in Health and Disease. 2019;18(1):1-11.
44. Usharani P, Mateen AA, Naidu MU, Raju YS, Chandra N. Effect of NCB-02, atorvastatin and placebo on endothelial function, oxidative stress and inflammatory markers in patients with type 2 diabetes mellitus: a randomized, parallel-group, placebo-controlled, 8-week study. Drugs in R&D. 2008;9(4):243-50.
45. Vanaie A, Shahidi S, Iraj B, Siadat ZD, Kabirzade M, Shakiba F, et al. Curcumin as a major active component of turmeric attenuates proteinuria in patients with overt diabetic nephropathy. Journal of Research in Medical Sciences. 2019;24:77.
46. Yang YS, Su YF, Yang HW, Lee YH, Chou JI, Ueng KC. Lipid-lowering effects of curcumin in patients with metabolic syndrome: a randomized, double-blind, placebo-controlled trial. Phytotherapy Research. 2014;28(12):1770-7.
