# Supplementary material for: Effects of turmeric (Curcuma longa) supplementation on glucose metabolism in diabetes mellitus and metabolic syndrome: An umbrella review and updated meta-analysis
Source: PLoS One. 2023 Jul 20;18(7):e0288997. doi: 10.1371/journal.pone.0288997 (PMC10359013; doi:10.1371/journal.pone.0288997)
Supplement: S1 File — (ZIP) [file pone.0288997.s002.zip › Table S8.pdf]

Table S8. Difference in changes of each outcome within 4 months between *Curcuma longa* supplementation and control group.

Outcomes	Post-intervention value						Change from baseline					
	Trials (n)	Control (n)	Curcumin (n)	Mean difference (95% CI)	P value	Heterogeneity (I^2)	Trials (n)	Control (n)	Curcumin (n)	Mean difference (95% CI)	P value	Heterogeneity (I^2)
FBG (mg/dL)	23	979	1019	**–8.129 (–12.175, –4.084)**	**<0.001**	75.8%	14	482	557	**–8.833 (–13.907, –3.758)**	**<0.001**	98.2%
HbA1C (%)	21	896	936	–0.134 (–0.304, 0.037)	<0.001	83.0%	11	389	424	**–0.517 (–0.707, –0.327)**	**0.004**	61.3%
HOMA-IR (%)	12	582	597	0.137 (–0.941, 1.214)	0.804	99.0%	6	199	211	**–0.444 (–0.750, –0.139)**	**0.004**	96.5%
Insulin (μIU/mL)	12	549	584	**–0.663 (–1.156, –0.171)**	**0.008**	90.5%	6	199	211	**–0.686 (–0.890, –0.481)**	**<0.001**	47.1%
BMI (kg/m^2)	17	674	709	–0.328 (–1.093, 0.436)	0.400	87.5%	12	406	478	**–0.585 (–1.006, –0.164)**	**0.006**	99.0%
TC (mg/dL)	17	658	693	–1.818 (–12.854, 9.217)	0.747	95.7%	13	400	492	–5.597 (–12.182, 0.989)	0.096	99.5%
TG (mg/dL)	21	797	834	–4.899 (–12.980, 3.181)	0.235	88.1%	13	419	488	**–12.652 (–20.066, –5.238)**	**0.001**	91.4%
LDL-C (mg/dL)	20	774	806	**–6.199 (–12.061, –0.336)**	**0.038**	91.7%	14	438	530	–5.301 (–10.911, 0.310)	0.064	98.5%
HDL-C (mg/dL)	20	774	806	**2.746 (0.875, 4.617)**	**0.004**	90.8%	14	438	530	2.469 (0.538, 4.400)	0.012	98.9%
SBP (mm Hg)	10	391	401	–1.589 (–4.572, 1.394)	0.296	93.1%	6	195	244	–0.919 (–5.604, 3.766)	0.701	98.8%
DBP (mm Hg)	9	363	376	**–2.876 (–4.919, –0.833)**	**0.006**	87.7%	6	195	244	–1.960 (–5.351, 1.431)	0.257	99.2%
CRP (mg/L)	1	63	63	**–14.000 (–20.658, –7.342)**	**<0.001**	–	1	19	42	0.450 (–0.031, 0.931)	0.067	–
hs-CRP (mg/L)	10	329	339	–0.845 (–1.726, 0.036)	0.060	97.3%	7	259	312	**–0.589 (–1.158, –0.021)**	**0.042**	97.0%
uric acid (mg/dL)	2	125	121	–0.445 (–2.424, 1.533)	0.659	98.8%				NA		
IL-6 (pg/mL)				NA						NA		

Abbreviations: BMI, body mass index; CRP, C-reactive protein; DBP, diastolic blood pressure; FBG, fasting blood glucose; HbA1C, hemoglobin A1C; HDL-C, high-density lipoprotein cholesterol; HOMA-IR, homeostatic model assessment for insulin resistance; hs-CRP, high sensitivity C-reactive protein; IL-6, interleukin 6; LDL-C, low-density lipoprotein cholesterol; NA, not applicable; SBP, systolic blood pressure; SCr, serum creatinine; TC, total cholesterol; TG, triglyceride.
