# Supplementary material for: Effects of turmeric (Curcuma longa) supplementation on glucose metabolism in diabetes mellitus and metabolic syndrome: An umbrella review and updated meta-analysis
Source: PLoS One. 2023 Jul 20;18(7):e0288997. doi: 10.1371/journal.pone.0288997 (PMC10359013; doi:10.1371/journal.pone.0288997)
Supplement: S1 File — (ZIP) [file pone.0288997.s002.zip › Table S9.pdf]

Table S9. Subgroup analysis of difference in change of fasting blood glucose (mg/dL) within 4 months between *Curcuma longa* supplementation and control group.

Outcomes	Post-intervention value						Change from baseline					
	**Trials (n)**	**Control (n)**	**Curcumin (n)**	**Mean difference (95% CI)**	**P value**	**Heterogeneity (I^2)**	**Trials (n)**	**Control (n)**	**Curcumin (n)**	**Mean difference (95% CI)**	**P value**	**Heterogeneity (I^2)**
Baseline BMI												
• < 30 kg/m2	19	838	857	**–8.832 (–12.974, –4.691)**	**<0.001**	74.5%	10	363	375	**–11.055 (–15.808, –6.302)**	**<0.001**	85.2%
• ≥ 30 kg/m^2	2	61	82	–19.590 (–51.629, 12.448)	0.231	82.8%	3	99	162	–3.253 (–9.468, 2.962)	0.305	76.9%
Baseline TC												
• < 200 mg/dL	10	410	431	**–8.170 (–13.293, –3.047)**	**0.002**	61.8%	5	171	170	–5.512 (–11.796, 0.773)	0.086	0.0%
• ≥ 200 mg/dL	7	269	292	**–11.041 (–19.337, –2.746)**	**0.009**	64.6%	6	205	284	–9.245 (–19.235, 0.744)	0.070	98.0%
Baseline TG												
• < 150 mg/dL	5	140	156	**–11.576 (–21.230, –1.921)**	**0.019**	80.5%	2	60	81	–0.150 (–0.528, 0.229)	0.439	0.0%
• ≥ 150 mg/dL	15	655	680	–5.516 (–11.555, 0.523)	0.073	75.0%	10	354	411	**–10.077 (–17.245, –2.910)**	**0.006**	85.5%
Baseline LDL-C												
• < 100 mg/dL	5	196	194	–9.217 (–32.495, 14.060)	0.438	84.2%	3	106	104	–5.849 (–17.100, 5.403)	0.308	21.5%
• ≥ 100 mg/dL	14	576	614	**–7.214 (–12.189, –2.240)**	**0.004**	77.6%	9	308	388	**–8.635 (–16.879, –0.391)**	**0.040**	96.8%
Baseline HDL-C												
• < 45 mg/dL	12	447	466	**–7.709 (–13.289, –2.128)**	**0.007**	72.3%	7	243	260	**–12.612 (–20.277, –4.947)**	**0.001**	80.6%
• ≥ 45 mg/dL	7	325	342	–7.061 (–18.081, 3.958)	0.209	83.0%	5	171	232	–0.157 (–0.535, 0.220)	0.414	0.0%
Baseline FBG												
• < 130 mg/dL	7	303	329	**–6.560 (–10.060, –3.059)**	**<0.001**	52.9%	4	124	188	–0.162 (–0.539, 0.216)	0.402	0.0%

Outcomes	Post-intervention value						Change from baseline					
	**Trials (n)**	**Control (n)**	**Curcumin (n)**	**Mean difference (95% CI)**	**P value**	**Heterogeneity (I^2)**	**Trials (n)**	**Control (n)**	**Curcumin (n)**	**Mean difference (95% CI)**	**P value**	**Heterogeneity (I^2)**
• ≥ 130 mg/dL	16	676	690	**–8.398 (–15.574, –1.222)**	**0.022**	76.3%	10	358	369	**–12.846 (–17.918, –7.774)**	**<0.001**	85.5%
Baseline HbA1C												
• < 7%	6	285	307	**–8.457 (–11.528, –5.386)**	**<0.001**	34.7%	4	149	174	–10.978 (–23.722, 1.765)	0.091	88.0%
• ≥ 7%	15	611	629	**–8.559 (–15.837, –1.281)**	**0.021**	77.7%	7	240	250	**–11.408 (–16.840, –5.976)**	**<0.001**	88.4%
Baseline SBP												
• < 130 mmHg	9	326	347	**–3.799 (–6.924, –0.675)**	**0.017**	0.0%	4	113	150	–4.992 (–13.476, 3.491)	0.249	79.0%
• ≥ 130 mmHg	5	221	234	–6.191 (–18.469, 6.087)	0.323	79.4%	3	107	119	**–21.823 (–30.896, –12.750)**	**<0.001**	44.2%
Baseline DBP												
• < 80 mmHg	8	318	337	–2.108 (–7.118, 2.902)	0.410	36.9%	3	86	83	**–10.000 (–10.787, –9.213)**	**<0.001**	0.0%
• ≥ 80 mmHg	6	229	244	**–9.250 (–17.509, –0.992)**	**0.028**	70.6%	4	134	186	**–14.060 (–27.156, –0.964)**	**0.035**	93.0%
Trial included patient												
• with DM	17	664	679	**–7.829 (–14.549, –1.109)**	**0.022**	78.3%	8	275	285	**–11.384 (–16.575, –6.192)**	**<0.001**	86.8%
• without DM	6	315	340	**–7.575 (–11.488, –3.662)**	**<0.001**	55.0%	6	207	272	–5.763 (–12.251, 0.724)	0.082	77.1%
• with pre-DM	4	218	238	**–5.459 (–10.730, –0.189)**	**0.042**	75.6%	1	19	42	–0.152 (–0.531, 0.227)	0.431	–
• without pre-DM	19	761	781	**–8.673 (–14.426, –2.921)**	**0.003**	73.9%	13	463	515	**–10.029 (–14.445, –5.612)**	**<0.001**	84.8%
• with MetS	5	189	202	**–8.944 (–16.917, –0.972)**	**0.028**	82.1%	6	199	252	**–10.850 (–20.858, –0.842)**	**0.034**	89.8%
• without MetS	18	790	817	**–7.626 (–12.685, –2.567)**	**0.003**	71.6%	8	283	305	**–7.134 (–13.511, –0.758)**	**0.028**	98.6%
Curcumin product												

Outcomes	Post-intervention value						Change from baseline					
	**Trials (n)**	**Control (n)**	**Curcumin (n)**	**Mean difference (95% CI)**	**P value**	**Heterogeneity (I^2)**	**Trials (n)**	**Control (n)**	**Curcumin (n)**	**Mean difference (95% CI)**	**P value**	**Heterogeneity (I^2)**
• Whole compound	6	187	203	–6.320 (–15.850, 3.210)	0.194	81.2%	4	111	122	–9.745 (–21.093, 1.604)	0.092	83.0%
○ Dose < 1,500 mg/day				NA			1	40	40	–2.060 (–9.918, 5.798)	0.607	86.4%
○ Dose ≥ 1,500 mg/day	6	187	203	–6.320 (–15.850, 3.210)	0.194	81.2%	3	71	82	**–15.438 (–23.633, –7.243)**	**<0.001**	40.3%
• Extraction product	9	477	500	**–6.865 (–11.519, –2.211)**	**0.004**	61.4%	4	131	129	**–9.959 (–10.743, –9.175)**	**<0.001**	0.0%
○ Dose < 1,000 mg/day	4	134	158	–6.526 (–16.298, 3.247)	0.191	62.9%	2	71	71	–6.317 (–17.741, 5.107)	0.278	0.0%
○ Dose ≥ 1,000 mg/day	5	343	342	**–6.513 (–12.807, –0.218)**	**0.043**	62.6%	2	60	58	**–9.976 (–10.762, –9.190)**	**<0.001**	0.0%
• Bioavailability-enhanced Extract	8	315	316	**–13.104 (–25.547, –0.661)**	**0.039**	81.0%	7	280	306	**–6.666 (–12.889, –0.442)**	**0.036**	83.2%

Abbreviations: BMI, body mass index; CL, *Curcuma longa*; DBP, diastolic blood pressure; DM, diabetic mellitus; FBG, fasting blood glucose; HbA1C, hemoglobin A1C; HDL-c, high-density lipoprotein cholesterol; LDL-c, low-density lipoprotein cholesterol; MetS, metabolic syndrome; NA, not applicable; pre-DM, pre-diabetic mellitus; SBP, systolic blood pressure; TC, total cholesterol; TG, triglyceride.
