# Supplementary material for: Effects of turmeric (Curcuma longa) supplementation on glucose metabolism in diabetes mellitus and metabolic syndrome: An umbrella review and updated meta-analysis
Source: PLoS One. 2023 Jul 20;18(7):e0288997. doi: 10.1371/journal.pone.0288997 (PMC10359013; doi:10.1371/journal.pone.0288997)
Supplement: Attachment — Submitted filename: 02_Rebuttal letter_20230529.docx [file pone.0288997.s003.docx]

Dear editor,

Thank you for your valuable recommendation and comments. The revisions were summarized as follows,

Reviewer #2,

| **No.** | **comments** | **Original version**  (Line no. in the original version) | **Edit or Change**  (Line no. in the revised version) |
| --- | --- | --- | --- |
|  | The only minor flaw in this manuscript is the lack of an expansion discussion regarding the reasons and mechanisms of the effect of turmeric on reducing glycemic indices. | **This was mention in discussion 2^nd^ paragraph line 344-360** |  |
|  | **Title** |  |  |
| 1 | Title change to Effects of turmeric (Curcuma longa) supplementation on glucose metabolism in diabetes mellitus and metabolic syndrome: an umbrella review and updated meta-analysis. | Lines 1–2,  Effects of *Curcuma longa* supplementation on glucose metabolism in diabetes mellitus and metabolic syndrome: an umbrella review and updated meta-analysis | The title was changed as the reviewer recommended.  Lines 1–2,  Effects of **turmeric (*Curcuma longa*)** supplementation on glucose metabolism in diabetes mellitus and metabolic syndrome: an umbrella review and updated meta-analysis |
|  | **Abstract** |  |  |
| 2 | Line 20: Curcuma longa convert to turmeric (Curcuma longa) | Line 20,  “…(RCTs) of turmeric *Curcuma longa* (CL) and its related compounds…” | The common name was added as recommended.  Line 20,  “…(RCTs) of **turmeric** **(*Curcuma longa*, CL)** and its related bioactive compounds…” |
| 3 | Lines 20 & 21: "its related compounds on glycemic" convert to "its related bioactive compounds on glycemic" | Line 20–21,  “…and its related compounds on glycemic…” | The sentence was changed as the reviewer recommended.  Line 20–21,  “…and its related **bioactive** compounds on glycemic…” |
| 4 | Line 22 and the remainder of abstract: CL convert to turmeric | – | We appreciate your advice. We acknowledge turmeric is a well-known common name of *Curcuma longa* L. However, using the turmeric term could mislead many plants, such as, *C. inodora* Blatt., *C. pseudomontana* J.Graham, *C. caulina* J.Graham, *C. roscoeana* Wall., and *Zingiber officinale* Roscoe [1]. Therefore, to prevent common taxonomical errors [2], we agree to preserve *Curcuma longa* and its abbreviation, CL, according to the original manuscript.  References:   1. POWO (2023). "Plants of the World Online. Facilitated by the Royal Botanic Gardens, Kew. Published on the Internet; <https://powo.science.kew.org/results?q=turmeric>. Retrieved 30 May 2023. 2. Bennett, B. C., & Balick, M. J. (2014). Does the name really matter? The importance of botanical nomenclature and plant taxonomy in biomedical research. Journal of Ethnopharmacology, 152(3), 387–392. |
| 5 | Lines 22 & 23: "different CL preparation-forms" convert to "different CL preparation forms" OR "different CL preparations" (Conversion should be accomplished throughout the text of the manuscript) |  | The words have changed as reviewer recommended from “CL preparation-forms” to “***CL preparation forms***” and checked throughout the manuscript. |
| 6 | Line 26 & 27: As the authors know, Scopus and Web of Science are big databases. Why did the authors not use these databases? The number of articles on a topic may be 3-4 times more in Scopus database than PubMed database. | Lines 26–27,  “…The MEDLINE, Embase, and The Cochrane Library databases were searched…” | We apologize for the typographical error. Scopus was one of the included databases according to our protocol. The abstract was revised and corrected.  Lines 26–27,  “…The MEDLINE, Embase, ***The Cochrane Central Register of Control Trials, and Scopus*** databases were searched…” |
|  | **Introduction** |  |  |
| 7 | Line 73. CL preparation forms convert to turmeric (CL) preparation forms | – | Please refer to the response to reviewer comments #4. |
| 8 | Line 79. "the effects of curcumin and its related compounds on glycemic" converted to "the effects of turmeric preparations and its related bioactive compounds on glycemic" | Line 79,  “…curcumin and its related compounds on glycemic…” | Line 79,  “…curcumin and its related **bioactive** compounds on glycemic…” |
|  | **Methods:** |  |  |
| 9 | In flowchart (Fig 1), the authors identified that 1750 abstracts were extracted from Scopus database. Why this database didn't mention in the abstract together the other databases? | Line 26-27,  “…The MEDLINE, Embase, and The Cochrane Library…” | The Scopus database was included in the systematic searching according to our study protocol. We followed the advice by correcting the abstract.  Line 26-27,  “…The MEDLINE, Embase, The Cochrane Central Register of Control Trials, **and Scopus databases**…” |
|  | **Types of interventions** |  |  |
| 10 | Line 115: "curcuminoid standardized extract" convert to "standardized curcuminoid extract" | Line 115,  “…e.g., a curcuminoid standardized extract; and 3) …” | Line 115,  “…e.g., a **standardized curcuminoid extract**; and 3)…” |
| 11 | Please kindly correct S1 Table to Table S1, S2 Table to Table S2, etc. throughout the manuscript text. | – | We followed the advice of the reviewer’s comments by correcting the label of supplementary table throughout the manuscript. |
|  | **Outcomes of interest** |  |  |
| 12 | According to NCEP ATP 111, Mets was identified based on five criteria. Among five criteria, there aren't serum uric acid and hsCRP. The authors describe, why these biomarkers selected for Mets outcomes. |  | We followed our study protocol. Although the exact mechanism has not yet been clarified., several evidence implied that an elevation of serum uric, C-reactive protein (CRP) and high sensitivity C-reactive protein (hs-CRP) are associated with an increased risk of developing metabolic syndrome components, such as hypertension, insulin resistance, and dyslipidemia [1-3]. With a certain number of published evidence and none of the previous SRMAs have been investigated related to these markers, therefore we included them as one of outcomes of interest to provide the most up-to-date and comprehensive evidence. We added more details to acknowledge this comment as below.  References:   1. López-Olivo MA, et al. Serum uric acid as a predictor of incident metabolic syndrome: a secondary analysis of a longitudinal cohort study. Arthritis Care Res. 2013;65(12):2026-2031. 2. Kanbay M, et al. Uric acid and metabolic syndrome: lessons from a large, multicenter study. PLoS ONE. 2018;13(3):e0194125. 3. Lin SD, et al. Hyperuricemia and metabolic syndrome: associations with endothelial dysfunction. Am J Med Sci. 2010;339(3):233-239. |
|  | **Data synthesis and statistical analysis** |  |  |
| 13 | Line 161: "lipid profiles" modify to "lipoprotein profiles" |  | We followed our study protocol. A lipid profile typically includes measurements of total cholesterol, high-density lipoprotein cholesterol (HDL-C), low-density lipoprotein cholesterol (LDL-C), and triglycerides,which are the common markers used in a routine clinical practice and also general clinical research. Whereas the term “lipoprotein profiles” may refer to subclasses of lipoproteins, such as, LDL particles (including small, dense LDL), very low-density lipoprotein (VLDL) particles, and HDL subclasses. To provide the clinical aspects for the readers, we agreed to preserve the term “lipid profiles” according to the original manuscript. |
|  | **Results** |  |  |
| 14 | As I noted in comment 11, all "S1, 2, 3, ....... Table" modify to "Table S1, 2, 3, ....... " In the manuscript text | – | The words were changed as reviewer recommended and checked throughout the manuscript. |
| 15 | The authors should be modified "S1, 2, 3, ...... Fig" to "Fig S1, 2, 3, ...... " throughout in manuscript text similar to Tables. | – | The words were changed as reviewer recommended and checked throughout the manuscript. |
|  | **Table 1:** |  |  |
| 16 | BMI is abbreviate body mass index not bone mass index. Modify it in footnote of Table 1. | Lines 224 and 313,  “Abbreviations: BMI, bone mass index;…” | We apologize for the typographical error. The word was corrected and checked throughout the manuscript.  Lines 223 and 310,  “Abbreviations: BMI, **body mass index**;…” |
| 17 | Would you please review Huang [19] study rows for biomarkers in table 1? Is there any inconsistency in recorded data in table 1? |  | We have rechecked all the records. All reported data is correct. The number of reported outcomes may difference within its original study because of the difference in number of subpopulations reported for each outcome. |
|  | **Figs:** |  |  |
| 18 | Did the professor Ammarin Thakkinstian perform all the meta-analyses of this study? | Lines 162-166,  “All analyses were performed using STATA® version 17.0 (StataCorp, College Station, Texas, USA) along with the self-programmed STATA® for network meta-analysis described elsewhere[32]. A p-value ≤ 0.05 was considered statistically significant.”  Line 513  32. Chaimani A, Higgins JP, Mavridis D, Spyridonos P, Salanti G. Graphical tools for network meta-analysis in STATA. PLoS One. 2013;8(10):e76654. | We added more details about the author’s responsibility for “Data synthesis and statistical analysis” as described below. We also corrected the method on STATA program and the citation error as below.  Lines 162-166,  “All analyses were performed **by P.J., T.P., and W.R.** using STATA® version 17.0 (StataCorp, College Station, Texas, USA) along with the self-programmed STATA® **for meta-analysis described elsewhere**[32]. **A.T. and S.N. provided technical analysis advice.** A p-value ≤ 0.05 was considered statistically significant.”  Line 513  32. **Palmer TM, Sterne JAC. Meta-analysis in Stata: An Updated Collection from the Stata Journal: Stata Press; 2016.** |
| 19 | Line 321: "bone mass index" modify to "body mass index" in Abbreviation of Fig 6. | Line 321,  “Abbreviations: BMI, bone mass index;…” | We apologize for the typographical error. The word was corrected.  Line 318,  “Abbreviations: BMI, **body mass index**;…” |
| 20 | Line 332: "S30-S42 Figs" modify to "Figs S30-S42". The other Tables and Figs should be corrected. | – | The words were changed as reviewer recommended and checked throughout the manuscript. |
| 21 | Lines 367-368: What are "bioavailability-enhanced preparations" in the other studies? Modify to "bioavailability-enhanced preparations such as | Lines 368,  “…bioavailability-enhanced preparations increased the absorption…” | We followed the advice of the reviewer’s comments by adding the details to explain about the preparation.  Lines 365–367,  “…bioavailability-enhanced preparations**,** **such as curcumin-phosphatidylcholine phytosome**  **complex, curcumin micelles, water-soluble curcumin formulation containing, or colloidal nanoparticle formulation,** increased the absorption…” |
| 22 | The last paragraph of the discussion section should be separated under "Limitations and Strengths". | Line 381,  “Our study has several limitations. First,…” | The last paragraph was separated as recommended.  Line 378,  “**Limitations and Strengths**  Our study has several limitations. First,…” |
| 23 | As the authors converted Type 2 diabetes mellitus to T2DM, metabolic syndrome modify to Mets in throughout the manuscript text. | – | The metabolic syndrome was converted to MetS throughout the manuscript. |
| 24 | Which of the tables or figures in the supporting information section are more important to appear in the manuscript? Or to remain in the supporting information section? |  | We have reviewed throughout the manuscript. All major results with graphic have been reported in main manuscript. |
