# Supplementary material for: Effects of turmeric (Curcuma longa) supplementation on glucose metabolism in diabetes mellitus and metabolic syndrome: An umbrella review and updated meta-analysis
Source: PLoS One. 2023 Jul 20;18(7):e0288997. doi: 10.1371/journal.pone.0288997 (PMC10359013; doi:10.1371/journal.pone.0288997)
Supplement: Attachment — Submitted filename: 02_Rebuttal letter_20230705.docx [file pone.0288997.s004.docx]

Dear editor,

Thank you for your valuable recommendation and comments. The revisions were summarized as follows,

Reviewer #2,

| **No.** | **comments** | **Original version**  (Line no. in the original version) | **Edit or Change**  (Line no. in the revised version) |
| --- | --- | --- | --- |
| 8 | “the effects of curcumin and its related compounds on glycemic” converted to “the effects of turmeric preparations and its related bioactive compounds on glycemic” | Line 79,  “…the effects of **curcumin** and its related bioactive compounds on glycemic…” | We followed the advice of the reviewer’s comments by modifying the following sentences.  Line 79,  “…the effects of **turmeric preparations** and its related bioactive compounds on glycemic…” |
| 12 | “Outcomes related to MetS were also collected …” should be modified to “The other selected outcomes were also collected …”  Based on the response to comment 21, comment 12 should be modified! On the other hand, unfortunately, the authors did not understand my comment (number 12). Some biomarkers were measured for participants took part in a study either might exaggerated some main biomarkers and patients outcome Or they are measured according to the wishes of the authors. Therefore, authors should be subject to the reviewer's comments. If the authors want measure association metabolic syndrome with C-peptide; we can assume that this indicator is one of the main indicators of metabolic syndrome? Such an assumption is completely wrong. Researchers can measure the correlation/association/relationship of a biomarker or an outcome in a disease based on a strong implementation necessity, but they cannot relate that biomarker as a main indicator to that disease. | "Line 126,  “…Outcomes related to MetS were also collected including…”" | We followed the advice of the reviewer’s comments by modifying the following sentences.  Line 126,  “…The other selected outcomes were also collected including…” |
| 13 | The authors mentioned that they followed the study protocol. This explanation was rejected. If a phrase or phrases was/were wrong, we could not compliance with it/them. Hence, the phrases should be modified to “lipid and lipoprotein profile”. | Line 160,  “…baseline characteristics of patients (including mean body mass index [BMI], **lipid profiles**, blood pressure…” | We followed the advice of the reviewer’s comments by modifying the following sentences.  Line 160,  “…baseline characteristics of patients (including mean body mass index [BMI], **lipid and lipoprotein profiles**, blood pressure…” |
| 17 | I am not convinced.  Would you please review Huang [19] study rows for biomarkers in table 1? Is there any inconsistency in recorded data in table 1? |  | We have gone through this paper and have changed numbers to be consistent with the original paper. Please accept our sincere apology for our mistake. |
| 19 | Bone mass index (BMI) was not a typographical error. It was a wrong compliance. |  | We apologize for our inaccurate explanation of our response. It was a wrong compliance. The manuscript has been corrected according to the reviewer’s comments. |
| 24 | The authors didn't understand what I meant. I mean, which of the tables and figures in the appendix section should be added to the text of the main manuscript? |  | After careful consideration, we believe that the information included in the main manuscript is sufficient to maintain concise and focused data according to the objective of study. We also cited in the main manuscript to refer related additional information to the supplementary.  In case you feel that we still do not fully understand your question well enough, we kindly request you to clearly specify those details. We will thoroughly review your suggestions and make necessary revisions accordingly. Thank you. |
